# Unravelling the Influence of Apolipoprotein A5 Gene Polymorphisms on Lipid Profiles of Statin-Treated Patients: A Scoping Review

**DOI:** 10.21315/mjms-04-2026-240

**Published:** 2026-06-30

**Authors:** Thachainy Vadeevellu, Noraida Hassan, Hamid Jan Jan Mohamed, Nur Salwani Bakar

**Affiliations:** 1 School of Health Sciences, Health Campus, Universiti Sains Malaysia, 16150 Kubang Kerian, Kelantan, Malaysia; 2 Hamdan Tahir Library, Health Campus, Universiti Sains Malaysia, 16150 Kubang Kerian, Kelantan, Malaysia

**Keywords:** apolipoprotein A5, single nucleotide polymorphism, statins, triglyceride, high-density lipoproteins

## Abstract

The apolipoprotein A5 (APOA5) protein plays a critical role in lipid metabolism, particularly in the regulation of triglycerides (TG) and high-density lipoprotein cholesterol (HDL-C). Given the scattered nature of existing data, this review aims to consolidate current knowledge on *APOA5* gene polymorphisms and their effects on lipid profiles among statin users. Using Arksey and O’Malley’s framework, a systematic search was performed encompassing studies published between April 2002 and April 2026. From 617 titles initially identified, 14 studies met the inclusion criteria and were reviewed narratively by two independent reviewers. Two single nucleotide polymorphisms (SNPs) in the *APOA5* gene, rs662799 (c.-1131T>C) and rs3135506 (c.56C>G), were consistently associated with TG and HDL-C levels in individuals receiving statin monotherapy or combination lipid-lowering treatment. Carriers of the minor alleles exhibited higher baseline and on-treatment TG levels, accompanied by lower HDL-C, indicating a greater predisposition to hypertriglyceridaemia (HTG). The influence of these variants was more pronounced before statin initiation, while post-treatment associations were variable. Evidence for the impact of *APOA5* polymorphisms on low-density lipoprotein cholesterol (LDL-C) was limited, suggesting that statin-induced LDL-C reduction may override genetic effects. Additionally, *APOA5* variants have been linked to differential lipid responses to selected plant-derived lipid-lowering agents. Polymorphisms in the *APOA5* gene appear to modulate TG and HDL-C levels significantly, but not LDL-C, highlighting their potential utility as biomarkers for hyperlipidaemia and personalised lipid-lowering strategies in statin-treated individuals. Further functional and metabolomic studies are needed to clarify their mechanistic roles.

## Introduction

Apolipoproteins (APOs) are integral to lipoprotein metabolism, serving as structural components of lipoprotein particles, enzyme cofactors and ligands for cell-surface receptors (1). The apolipoprotein A5 (APOA5) protein, predominantly synthesised in the liver, is found on triglyceride (TG)-rich lipoproteins (which include very-low-density lipoprotein, VLDL, and chylomicrons) and high-density lipoproteins (HDLs) in the plasma (2). The *APOA5* gene is part of a regulatory gene cluster that includes *APOA1*, *APOC3* and *APOA4* (3). It is located on chromosome 11q23, approximately 30 kb downstream of *APOA4*, and it comprises four exons that encode a protein of 369 amino acids (4). The APOA5 protein plays a key role in modulating plasma TG levels in both humans and mice, primarily by promoting TG hydrolysis (4, 5). Although the precise mechanisms remain unclear, the APOA5 protein is believed to enhance the activity of lipoprotein lipase (LPL), thereby accelerating the catabolism of TG-rich lipoproteins. It may also interact with members of the low-density lipoprotein receptor (LDLR) family to facilitate the uptake of remnant lipoprotein particles (6). Genetic polymorphisms in *APOA5* can disrupt its function, potentially resulting in elevated TG levels (7) and contributing to hypertriglyceridaemia (HTG) (4). In fact, genome-wide association studies have identified associations between *APOA5* polymorphisms and plasma TG variability across different Southeast Asian populations (8). Additionally, evidence from clinical and basic research indicates that common single nucleotide polymorphisms (SNPs) in the *APOA5* gene have been associated with variations in TG levels (5).

Previous research demonstrated that mice engineered with the human *APOA5* transgene exhibit significantly lower plasma TG levels, whereas *APOA5* knockout mice lacking the APOA5 protein show TG levels approximately four times higher than those of control mice (9). These findings underscore the pivotal role of *APOA5* in lipid regulation, particularly TG metabolism, positioning it as a strong candidate gene for assessing coronary artery disease (CAD) risk. Plasma TG levels are influenced by both genetic and environmental factors, with genetic predisposition being the primary contributor to HTG (10–12). Although high TG levels are associated with increased CAD risk, the underlying mechanisms remain unclear (11).

While no specific SNP in the *APOA5* gene has been definitively linked to specific lipid responses or disease outcomes, growing evidence indicates that SNPs such as *APOA5* c.-1131 T>C (rs662799) and c.56C>G (rs3135506) are associated with elevated TG levels (13, 14), as well as other lipid profiles such as HDL-C, non-HDL-C and total cholesterol levels in Caucasians (15). However, a comprehensive understanding of the underlying mechanisms remains incomplete, and existing data on these polymorphisms are fragmented, especially among statin users. Therefore, this scoping review seeks to consolidate current evidence on the association between *APOA5* polymorphisms and lipid profiles, along with their clinical relevance, among statin-treated patients. Statins are a first-line drug class used for managing dyslipidaemia and reducing cardiovascular risk.

## Methods

This scoping review included published gene association studies investigating the relationship between *APOA5* polymorphisms and lipid profiles in patients during statin treatment, while also incorporating evidence related to other lipid-lowering agents frequently employed in clinical practice.

### Search Strategy

This study adopted a scoping review design based on Arksey and O’Malley’s guidelines (16). These guidelines consist of five key stages: formulating research questions, identifying relevant studies, selecting studies, charting the data, and synthesising and reporting the findings. A comprehensive search was conducted across electronic databases including PubMed Clinical Queries, PubMed and MEDLINE, the Cochrane Library, EBSCOhost-CINAHL, ProQuest and ScienceDirect, covering publications from April 2002 to April 2026. The main Medical Subject Headings terms used in the search are detailed in Table 1. Only studies deemed potentially relevant to the study objectives were retrieved and thoroughly reviewed. The specific search categories used for the standard query, as outlined in Table 1, varied depending on the database and are described in detail in Supplementary File 1.

### Study Selection

#### Inclusion Criteria

The inclusion criteria for this review included: i) full-text articles published between April 2002 and April 2026 reporting cohort studies, case-control studies (prospective or retrospective), clinical trials, genome-wide linkage studies, Mendelian randomisation studies or evidence-based studies involving human; ii) articles written in, or translated into, English; iii) studies involving participants of all ages, genders, ethnicities and geographic locations; and iv) genetic association studies examining any SNP within or near the *APOA5* gene, with a focus on individuals receiving statin monotherapy, statins in combination with other lipid-lowering medications, or plant-derived lipid-lowering agents.

#### Exclusion Criteria

The exclusion criteria for this review included: i) review articles, roundtable discussions, editorials, conference abstracts, letters, commentaries, posters, dissertations or theses and book chapters; and ii) studies published before April 2002 or after April 2026.

#### Collating, Summarising and Reporting the Results

Study selection was guided by the research objectives and predefined inclusion criteria. The Preferred Reporting Items for Systematic Reviews and Meta-Analyses (PRISMA) flow diagram (17) was employed to document and organise the selection process. Two reviewers independently screened the articles and extracted relevant data. The search and study-selection outcomes were recorded using the PRISMA flow diagram (Figure 1), and data from eligible studies were discussed, tabulated and summarised. Table 2 provides a brief overview of the association between *APOA5* polymorphisms and the clinical parameters investigated in the included studies. The reviewers collaboratively analysed and synthesised the findings to ensure an accurate interpretation of the data.

## Results

### Characteristics of the Included Studies

The initial literature search identified 617 articles, of which only 14 met the inclusion criteria and were incorporated into this review, as shown in Table 2. Across the included studies, the minor allele frequencies (MAFs) of *APOA5* polymorphisms varied considerably among populations. Rare variants in European-American cohorts were observed at very low frequencies (MAF = 0.003–0.004), whereas common variants such as rs662799 showed moderate frequencies in Western populations (MAF = 0.05 in the United States and MAF = 0.08–0.10 in Czech and Brazilian cohorts) and markedly higher frequencies in Asian populations, including Chinese cohorts (MAF = 0.267–0.330) and a Malaysian population (MAF = 0.450) (Table 2).

The *APOA5* rs662799 and rs3135506 emerged as the most prominent SNPs, demonstrating strong associations with TG levels (18, 24–28) compared with other SNPs within or near the *APOA5* gene, such as rs651821, rs3135507 and rs2266788. Although replication data from gene association studies remain limited, other SNPs, such as rs964184 (a SNP in the *ZPR1* gene, which is located near the *APOA1-C3-A4-A5* gene cluster on chromosome 11) and *APOA5* rs2075291, have also been identified as key determinants of TG levels and the progression of metabolic syndrome in both human and animal studies (20–22, 32).

While *APOA5* polymorphisms have shown a consistent association with TG levels, studies in humans have also reported their influence on HDL-C, often indicating a negative correlation (19, 22). Specifically, SNPs such as rs3741298, rs694184, rs651821 and rs10750097 in the *APOA5-ZNF259* region were significantly associated with greater percentage changes in HDL-C level in response to statin and fenofibric acid (FA) therapy (*P* < 0.0001) (22). In contrast, a variant in the promoter region of *APOA5*, rs662799, was linked to either a reduction or no significant change in HDL-C levels following treatment with FA (18). Additionally, *APOA5* rs662799 did not appear to influence TG, HDL-C and low-density lipoprotein cholesterol (LDL-C) responses to statin therapy in Chinese patients, although it was associated with baseline HDL-C levels (24). This finding was consistent with that of another study involving Asian participants receiving statin (31).

Regarding the effects on LDL-C, there is limited evidence to suggest an association between *APOA5* polymorphisms and LDL-C levels, with most studies finding that variants within the *APOA5* gene did not significantly affect LDL-C profiles (24, 26). Regardless of statin type and dosage, the wild-type genotype of *APOA5* rs662799, unlike that of *APOA5* rs3135507, was observed to significantly affect LDL-C response in patients with dyslipidaemia receiving statin therapy (*P* < 0.05). This suggests that individuals carrying the wild-type allele benefited more from statin treatment than carriers of the minor C allele of this SNP (25).

## Discussion

This review evaluated the influence of *APOA5* polymorphisms on lipid parameters and their clinical implications among statin-treated individuals. The key findings highlight *APOA5* rs662799 (c.-1131T>C) and, to a lesser extent, *APOA5* rs3135506 (c.56C>G) as the most clinically significant SNPs associated with high TG and low HDL-C levels (Figure 2), suggesting their potential role in increasing cardiovascular disease risk. Currently, evidence linking *APOA5* variants to LDL-C levels remains limited, and the lipid-lowering effects of pharmacological agents may surpass genetic influences in reducing LDL-C levels. Despite treatment with lipid-lowering drugs (rosuvastatin, gemfibrozil and fenofibrate), recent evidence has provided direct mechanistic support for *APOA5* dysfunction, as demonstrated by a rare frameshift mutation (c.111delC) identified in a Hispanic female presenting with HTG and low HDL-C levels (33). This type of mutation is indicative of a loss-of-function mechanism and supports a more direct pathogenic role of *APOA5* in lipid profiles.

Individuals carrying the minor C allele of the *APOA5* rs662799 often exhibit variability in serum TG levels, with consistent associations observed between this variant and elevated non-fasting plasma TG levels, as well as increased remnant lipoprotein particles (26–28). This association is significantly linked to an increased risk of myocardial infarction and CAD (34). The expression of *APOA5* influences plasma TG levels by modulating the lipolysis of TG-rich lipoproteins, possibly through binding with lipoproteins, endothelial proteoglycans and LPL, thereby stabilising the lipolytic complex (35). As a rate-limiting enzyme, LPL normally hydrolyses circulating TG-rich lipoproteins such as VLDL and chylomicrons. Genetic polymorphisms impair LPL activity, resulting in characteristically elevated plasma TG levels and reduced HDL-C (35) and LDL-C levels (35, 36). Overall, it can be suggested that genetic polymorphisms in *APOA5* directly affect LPL activity and, in turn, plasma TG levels.

Certain SNPs in the *APOA5* gene, particularly in the absence of lipid-lowering drugs, have been consistently associated with elevated plasma TG levels, leading to HTG. For instance, carriers of the minor allele for *APOA5* rs662799 and *APOA5* rs2075291 have shown a strong association with HTG in individuals undergoing stable antiretroviral therapy for human immunodeficiency virus (HIV) (26, 37). Similarly, in Chinese patients with and without familial hypercholesterolaemia, the *APOA5* rs662799 variant has been implicated in dyslipidaemia due to increased TG levels (24). The homozygous mutant genotype of *APOA5* rs3135506 has also been strongly associated with higher TG levels across various populations (14, 38, 39). Additionally, *APOA5* rs964184 has been shown to promote TG elevation, although it does not appear to be a predictor of vascular events in patients with clinically manifest vascular disease (20).

Elevated fasting plasma TG levels characterise HTG, although other lipoprotein abnormalities may also be present (40). Lifestyle factors such as obesity, sedentary habits and diabetes contribute to the progression of HTG; however, genetic variants like *APOA5* rs2075291 (c.553G>T) can exacerbate the condition (21). Loss-of-function mutations in the *APOA5* gene, particularly when combined with protease inhibitor therapy, have been associated with severe HTG and hyperlipidaemia in HIV-infected patients (41). For example, the Q97X mutation in this gene was identified as a cause of severe HTG in a consanguineous Chilean family, where mutant homozygotes showed significantly higher TG levels compared to heterozygotes (23). In this case, mutant homozygotes completely lost their ability to initiate lipolysis, resulting in marked TG elevation, while heterozygotes experienced partial APOA5 deficiency (42). Nevertheless, Dussaillant et al. (23) suggested that the phenotypic expression of this mutation may vary widely due to environmental factors and patients’ clinical conditions. Despite some conflicting findings, *APOA5* polymorphisms remain valuable biomarkers for both the diagnosis and clinical management of HTG.

Emerging pharmacogenetic evidence suggests that the efficacy of TG-lowering therapies may be influenced by *APOA5* polymorphisms, potentially through mechanisms involving protein degradation. A genome-wide association study conducted in Southeast Asians in 2017 identified that a missense variant, *APOA5* rs2075291, conferred approximately a 60% increased risk of CAD per copy of the risk allele, highlighting its potential utility in diagnosing HTG within Asian populations (8). However, findings regarding this SNP have been inconsistent, as it has also been associated with TG-lowering effects, particularly during treatment with lipid-lowering agents (21, 30). For example, carriers of the minor T allele of the *APOA5* rs2075291 SNP exhibited reduced TG levels in CAD patients treated with atorvastatin (21). Similar outcomes were observed in magnolol-treated transgenic mice, where minor T allele carriers demonstrated lower postprandial plasma TG levels and enhanced TG metabolism in cases of mild postprandial HTG (32), suggesting a protective effect of this SNP against CAD during pharmacological intervention.

Furthermore, other *APOA5* gene variants, namely rs662799, rs3135506 and rs964184, have demonstrated a significant association with TG reduction in response to FA therapy (27). However, no notable changes in TG levels were observed following rosuvastatin treatment between individuals with the wild-type genotype and carriers of the rs662799 variant (24). This may be attributed to the inability of wild-type rs662799 genotypes to activate LPL via the G185C protein (glycine at residue 185 of the APOA5 protein), thereby impairing TG hydrolysis (15). Conversely, individuals with the heterozygous TC genotype (carry one copy of the major T allele and one copy of the minor C allele) were reported to produce a partially functional APOA5 protein capable of partial TG hydrolysis and, despite carrying at least one minor C allele, achieved TG reduction (15). Furthermore, high intake of monounsaturated fatty acids among minor C allele carriers of rs662799 has been associated with increased resistance to TG elevation (43), suggesting an inverse relationship between dietary fat intake and plasma TG levels in these individuals.

Beyond their well-established role in TG regulation, *APOA5* polymorphisms have also been implicated in modulating HDL-C levels. Specifically, SNPs within the *APOA5-ZNF259* region, such as rs3741298, rs964184, rs651821 and rs10750097, have shown significant associations with enhanced HDL-C responses in individuals treated with a combination of statin and FA (22). The *ZNF259* gene (which overlaps with the *ZPR1* gene locus) is located near the *APOA5* and may influence *APOA5* expression through regulatory interactions, thereby impacting HDL-C metabolism (22). The minor alleles of these SNPs may be related to improved reverse cholesterol transport or enhanced maturation of HDL particles (44), a process in which APOA5 also plays a supporting role. In addition to *APOA5* polymorphisms, inter-individual variability in HDL-C responses has been linked to other genetic loci involved in statin pharmacodynamics and pharmacokinetics. For example, the rs646776 SNP, located in an intergenic region within the sortilin/cadherin EGF LAG seven-pass G-type receptor 2/proline-serine-rich coiled-coil protein 1 (*SORT1-CELSR2-PSRC1*) gene cluster, has been associated with higher HDL-C levels, particularly in females (45). Similarly, the rs2231142 SNP in the ATP-binding cassette subfamily G member 2 (*ABCG2*) gene has also been associated with higher HDL-C levels (31).

Inconsistent findings regarding the influence of *APOA5* polymorphisms on HDL-C levels have been reported. Although one study reported a strong link between *APOA5* rs662799 and lower HDL-C levels in patients with and without hypercholesterolaemia (24), other studies have presented conflicting results regarding this association (26, 31, 34). The rare promoter variant, *APOA5* rs662799, has been associated with a paradoxical decrease in HDL-C levels following FA therapy (18), possibly due to the inherently low baseline HDL-C levels observed in those with the homozygous mutant genotype (carrying two copies of the minor C allele) at this locus (46). During FA therapy, alterations in the gene’s promoter region may interfere with its activation and, when combined with suppressed gene expression, may limit FA’s effectiveness in raising HDL-C levels (18). Moreover, studies that associate *APOA5* rs662799 with reduced plasma HDL-C levels suggest that this variant may partially account for the decrease (26, 31, 47). Indeed, a recent meta-analysis found no link between *APOA5* rs3135506 and HDL-C levels in response to FA therapy (14), consistent with the earlier findings during sterol treatment (48), where *APOA5* rs3135506 variants did not influence HDL-C levels, although sterols effectively reduced LDL-C levels (36, 48). These varying results highlight the need for more consistent and comprehensive studies to better understand the role of *APOA5* polymorphisms in HDL-C modulation.

Despite inconsistent findings regarding TG and HDL-C levels, the influence of *APOA5* polymorphisms on LDL-C levels appears to be variable and difficult to predict. Notably, mutant APOA5 proteins have been reported to exhibit a stronger binding affinity for members of the LDLR family compared to LDL particles themselves (46). Consequently, if APOA5 competitively inhibits LDL binding to these receptors, the LDL-C lowering efficacy of statin therapy may be attenuated, particularly among individuals carrying the variant. For example, although the *APOA5* rs3135506 variant demonstrated a significant association with changes in total cholesterol levels (14), no association (*n* = 45, *P* = 0.682) was observed between this variant and LDL-C levels in a Spanish cohort undergoing sterol treatment (36). Conversely, a marginal association with LDL-C levels (*P* = 0.047) was observed in a larger Brazilian cohort of HIV-infected patients with dyslipidaemia (*n* = 614) (26). Furthermore, carriers of the minor C allele of *APOA5* rs662799 experienced significantly diminished LDL-C reduction following statin therapy (*P* < 0.005) compared to individuals with the wild-type genotype (25), suggesting that this polymorphism may hinder the LDL-C lowering effect of statins. Mechanistically, the pharmacodynamic action of statins would enhance LDLR expression on hepatocytes, thereby facilitating the clearance of LDL particles from the bloodstream (46).

Given that APOA5 plays a crucial role in lipid metabolism, its dysfunction due to genetic polymorphisms often leads to clinical consequences, primarily through alterations in plasma TG levels. Indeed, polymorphisms in the *APOA1-C3-A4-A5* cluster is recognised as a conventional risk factor for CAD, one of the most prevalent clinical outcomes linked to gene polymorphisms (47). Notably, a strong association exists between TG, SNPs in *APOA5* and CAD risk, a relationship that persists even after adjustment for LDL-C and HDL-C levels (48). Mechanistically, functional APOA5 protein enhances LPL activity to hydrolyse TG into free fatty acids, a clearance process that plays a critical protective role against the development of CAD (48) and other cardiovascular diseases (14, 21, 28, 49). Specific variants within the *APOA5* gene demonstrate distinct, population-dependent associations with cardiovascular risk. For example, the variant allele of the *APOA5* rs662799 was found at a higher frequency in patients with coronary heart disease compared to healthy controls (28), with the risk being particularly pronounced in males (28, 31). Additionally, *APOA5* rs2075291 has been proposed as a potential genetic marker for CAD due to its strong correlation with elevated TG levels and HTG (21). Similarly, *APOA5* rs3135506 has been linked to increased CAD susceptibility under both dominant and recessive genetic models in the Moroccan population (14), although inconsistent results have been reported in the French population for SNPs in *APOA5* (50). This association may be explained by population-specific differences in allele distribution, as the *APOA5* variant exhibits a higher MAF in Moroccan cohorts (0.071 in controls and 0.184 in CAD patients; *P* < 0.001) compared with French cohorts (0.048 in controls and 0.077 in coronary heart disease patients; *P* = 0.012) (14, 50).

More than two decades ago, a large cohort study from the Czech population identified that polymorphisms in *APOA5* resulted in TG elevation and served as a risk factor for mortality due to myocardial infarction (51). Beyond *APOA5*, a recent report found that significant TG elevation can also result from homozygous or biallelic loss-of-function mutations in other genes, such as *LPL, APOC2, LMF1* and *GPIHBP1* (52). Specifically, for the *APOA5* gene, the occurrence of rare homozygous genotypes for one or both SNPs (i.e., rs662799 and rs3135506) was notably higher in patients with myocardial infarction and metabolic syndrome than in healthy individuals (53, 54). Interestingly, although the rs964184 SNP in the *APOA5-A4-C3-A1* gene cluster is linked to increased TG levels, it has not been associated with vascular events such as ischaemic stroke (20). This suggests that genetic risk in ischaemic stroke progression may be influenced by gene-gene interactions, as observed in Han and Uighur populations (29). Overall, with regard to ischaemic stroke incidence, a meta-analysis indicated that individuals carrying the minor C allele of rs662799 have a 1.33- to 1.43-fold greater risk of ischaemic stroke compared with carriers of other *APOA5* variants (54).

A significant body of research has examined how *APOA5* polymorphisms influence the effectiveness of lipid-lowering therapies, particularly statins. However, findings on the relationship between *APOA5* variants and responses to statin drugs remain inconsistent. For example, individuals carrying the minor T allele of *APOA5* rs2075291 and those with the wild-type TT genotype of *APOA5* rs662799 demonstrated significant responses to atorvastatin, showing reductions in TG and LDL-C levels, respectively (21, 25). Similarly, in another study, patients with the *APOA5* rs2075291 variant who received atorvastatin experienced a notable decrease in total serum TG levels (*P* < 0.05) compared with those on placebo (55). However, Caucasian carriers of the *APOA5* rs662799 minor C allele (*n* = 187) did not exhibit LDL-C reduction following atorvastatin treatment, suggesting that wild-type individuals (TT genotype) responded more favourably than minor C allele carriers (25). No significant associations with the LDL-C profiles were observed for other *APOA5* variants, such as c.56C>G (rs3135506) and c.457G>A (rs3135507), or for variants in other genes involved in lipid metabolism, such as *APOE* (24, 25).

Beyond statins, the *APOA5* gene also influences the effectiveness of other lipid-lowering medications such as fibrates. Both randomised controlled trials and case-control studies support the finding that statin therapy, either alone or combined with fibrates, can lower TG levels in individuals with *APOA5* rs662799 and *APOA5* rs2075291 (18, 21). Research suggests that gene-specific variations in TG levels may be explained by quantile-dependent expressivity, a phenomenon where genetic effects are more pronounced at higher baseline TG concentrations, resulting in nonparallel TG reductions across genotypes. Under this model, certain gene variants, such as those in *APOA5*, are linked to higher baseline TG levels, particularly in individuals with greater adiposity (27). For instance, carriers of the minor C allele of *APOA5* rs662799 exhibited a stronger impact on plasma TG levels in obese individuals compared with lean individuals (27). When evaluating the impact of lipid-lowering therapies on TG levels in patients with dyslipidaemia, it is important to consider additional predictors of lipid metabolism, such as genetic variants in the *CETP* gene and demographic factors like gender (56), which may further modulate treatment outcomes.

Other lipid-lowering agents, such as niacin (vitamin B3, which is used as an antihyperlipidaemic agent at doses ranging from 1,500 to 3,000 mg/day orally), have been found to increase HDL-C levels while lowering TG and LDL-C levels (57). To improve patient adherence, niacin is often administered concurrently with laropiprant to mitigate its flushing side effect (58). One study found no significant association between the *APOA5* rs662799 variant and lipid response to the niacin-laropiprant combination (58), suggesting limited pharmacogenetic relevance for these drugs. In contrast, bexarotene, a drug used to treat cutaneous T-cell lymphoma, with HTG being the main reason for discontinuation, showed favourable pharmacogenetic interactions with the *APOA5* rs662799 variant. Specifically, individuals carrying the minor allele did not experience a significant rise in TG levels following treatment (27), suggesting a potentially protective effect in this genetically defined subgroup.

Finally, plant-derived compounds such as magnolol, extracted from *Magnolia officinalis*, and phytosterols have also been employed as lipid-lowering agents. Magnolol has demonstrated efficacy in reducing TG levels, while phytosterols are known to lower LDL-C (32, 36). Interestingly, the effectiveness of these agents appears to be modulated by specific *APOA5* gene polymorphisms. For example, magnolol significantly reduced postprandial plasma TG levels in transgenic knock-in mice carrying the *APOA5* rs2075291 variant, likely through the upregulation of LPL activity in 3T3-L1 pre-adipocyte cells (32). In contrast, individuals with the *APOA5* rs3135506 variant did not exhibit significant LDL-C reduction following treatment with phytosterols (59). The lipid-lowering efficacy of phytosterols may depend more on baseline LDL-C levels and other intrinsic genetic factors (60). These findings further support the notion that *APOA5* polymorphisms predominantly influence TG metabolism, with a comparatively limited role in LDL-C regulation.

## Conclusion

This review provides an analysis of the relationship between various *APOA5* polymorphisms and their influence on lipid profiles, particularly TG, HDL-C and LDL-C, in individuals undergoing treatment with statins and other lipid-lowering agents. Although the precise biological mechanisms underlying these associations remain to be fully elucidated, the emerging role of pharmacogenetics in modulating lipid responses offers a promising direction for future research. A deeper understanding of *APOA5* variants and their impact on lipid metabolism could enhance clinical decision-making and support the development of personalised therapeutic strategies for cardiovascular disease management.

## Supplementary Information



## Figures and Tables

**Figure 1:**
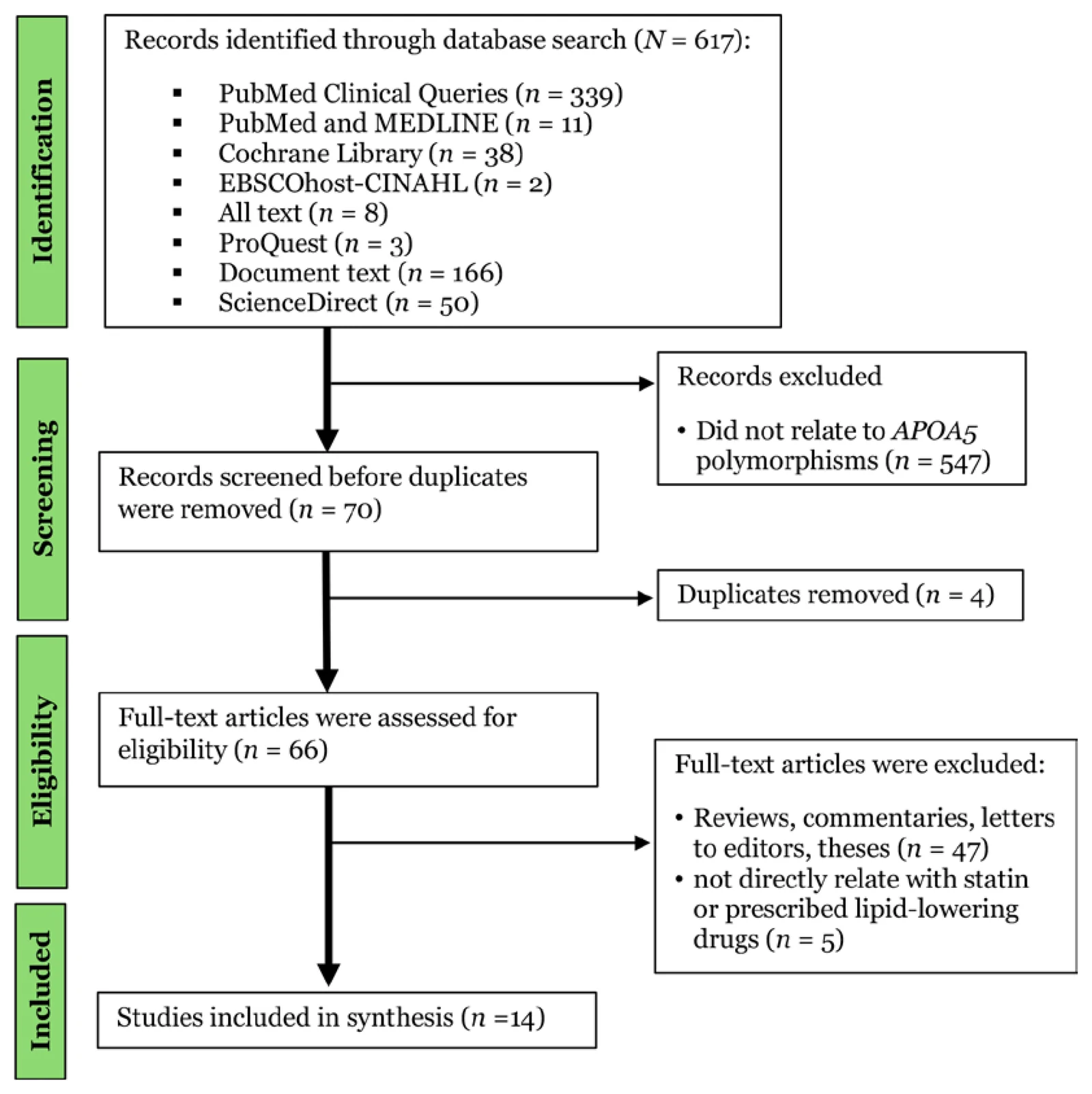
Identification and screening process in assessing eligible articles of this scoping review.

**Figure 2:**
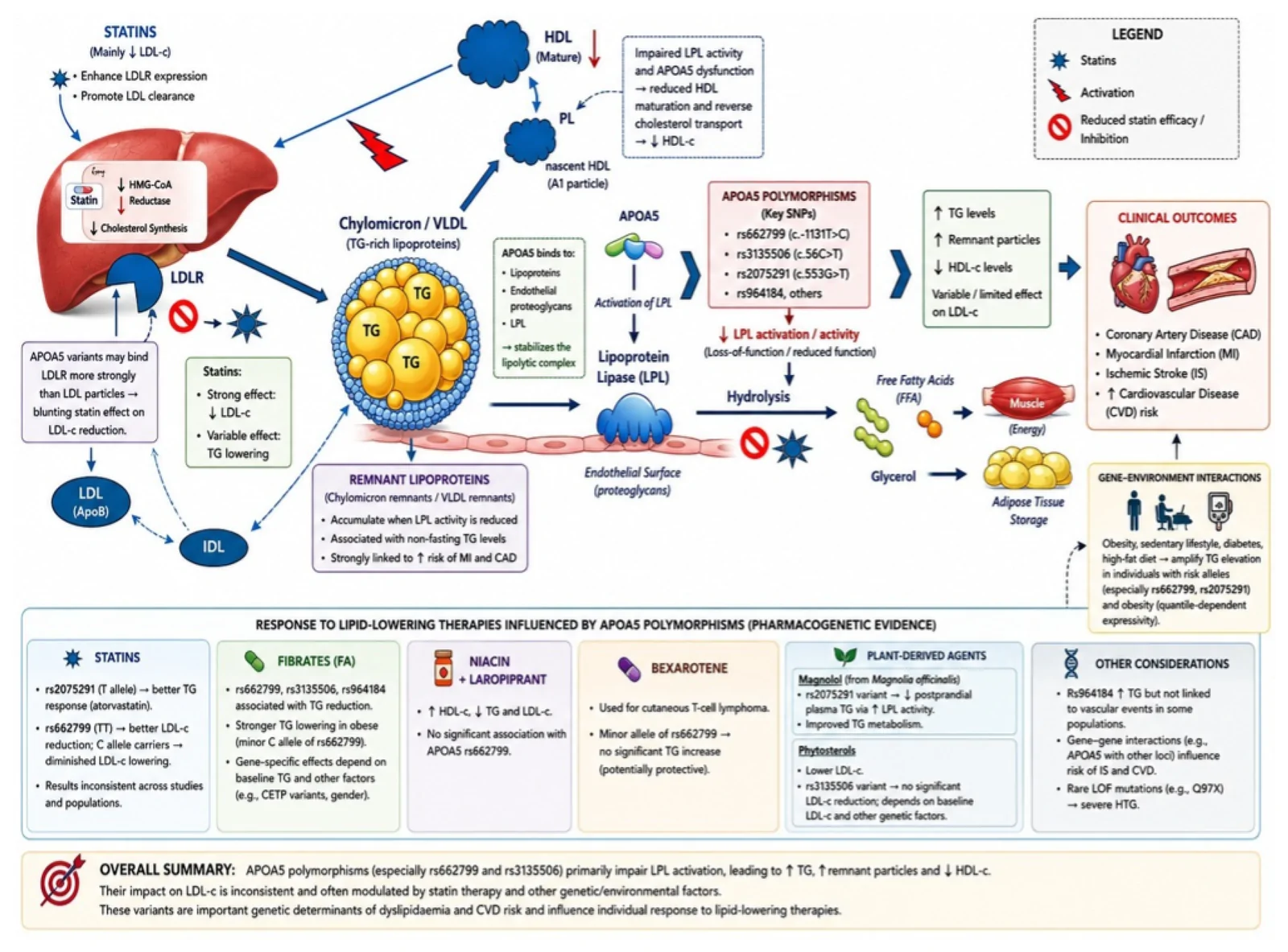
Impact of *APOA5* polymorphisms on lipoprotein metabolism, statin response, and cardiovascular risk. This figure illustrates the central role of APOA5 in regulating TG-rich lipoprotein metabolism via activation of LPL, and how genetic polymorphisms in *APOA5* impair this pathway, leading to elevated TG, reduced HDL-C levels, and the accumulation of remnant lipoproteins. In addition, the figure highlights the modulatory effect of these genetic variants on the efficacy of statins and other lipid-lowering therapies, thereby influencing cardiovascular disease risk through gene–environment interactions. APOA5 = apolipoprotein A5; ApoB = apolipoprotein B; TG = triglycerides; HDL = high-density lipoprotein; IDL = intermediate-density lipoprotein; LDL-C = low-density lipoprotein cholesterol; LPL = lipoprotein lipase; LDLR = LDL receptor; PL = phospholipid; VLDL = very-low-density lipoprotein. This figure was generated with AI-assisted support (ChatGPT, OpenAI) and finalised by the authors.

**Table 1:** Keywords used in the search strategy.

Search category	Query
#1	statin* OR “Hydroxymethylglutaryl-CoA Reductase Inhibitors” OR simvastatin OR atorvastatin OR rosuvastatin OR pravastatin OR lovastatin OR fluvastatin
#2	“APOA5 polymorphism” OR “APOA5 mutant” OR “APOA5 carrier” OR “APOA5 variant” OR “Apolipoprotein A-V” OR “Apolipoprotein A5” OR “Polymorphism, Single Nucleotide”
#3	“cholesterol lower*” OR “cholesterol reduction” OR “cholesterol decrease” OR cholesterol OR “LDL” OR “HDL”

**Table 2:** Overview of the studies included for data extraction, covering the period from April 2002 to April 2026.

Country	SNP(s) related to the *APOA5*	MAF	Study design Sample size and participant characteristics	Statin(s) used (alone or in combination)	Key findings
USA (European-American population) (18)	Rare variant in *APOA5*. i.e, *APOA5* promoter and *APOA5* missense variants	0.003–0.004	Randomised controlled study 1,612 individuals with mixed dyslipidaemia	FA alone and combination with statins	Rare variants in the *APOA5* promoter region were associated with reduced HDL-C and apoA-I levels in response to FA therapy; whereas rare *APOA5* missense variants were associated with increased TG response to combination therapy.
USA (Sacramento and Beltsville populations) (19)	*APOA5* rs662799 (c.-1131T>C)	0.050	Gene association study (65 SNPs from 23 genes) Sacramento population (*n* = 249) and Beltsville population (*n* = 532)	Not specify what type of statins (less than 7% for overall study population used statins)	The *APOA5* rs662799 was identified as a significant predictor for HDL-C levels, with the allelic substitution effect (regression slope) estimated at −4.523 in the Sacramento population and −0.471 in the Beltsville population, indicating a negative association between this SNP and HDL-C levels.
Netherland (20)	* rs964184 in the *ZPR1* gene (also refers to the *ZNF259* gene and it is located near the *APOA1-C3-A4-A5* gene cluster on chromosome 11)	0.142 (0.10–0.246, MAF increased with increased TG levels of the patients)	Prospective study 5547 patients with arterial disease	Majority of the patients were on statins, and a small proportion (0.8%) received fibrates	The minor allele of rs964184 was significantly associated with log-transformed plasma TG (*P* = 1.1 × 10^−19^), HDL-C (*P* = 0.007) and non-HDL-C levels (*P* = 6.3 × 10^−7^). The association between rs964184 and plasma TG was influenced by patients’ body mass index (*P* = 0.03). Homozygous recessive individuals exhibited a higher incidence of metabolic syndrome (*P* = 0.01). However, no association was observed between this SNP and the risk of new vascular events.
Saudi Arabia (21)	*APOA5* rs2075291 (c.553G>T)	Not specified	Case-control study 354 CAD patients, 377 controls	Atorvastatin	Among patients with CAD receiving atorvastatin therapy, carriers of the minor T allele of *APOA5* rs2075291 exhibited significantly lower TG levels (*P* = 0.001).
USA (European-American population) (22)	rs3741298, rs964184, rs651821 and rs10750097 (SNPs in the *APOA1-C3-A4-A5-ZPR1-BUD13* gene cluster)	0.18	Multicentre, randomised, double-blind, active controlled study 2,228 individuals with mixed dyslipidaemia	Combination therapy with statins and FA	The SNPs within the studied gene region exhibited markedly smaller genotype-related differences during fenofibrate/statin combination therapy when mean TG levels were low (1.64 mmol/L), compared with the larger pretreatment differences observed at higher mean TG levels (3.15 mmol/L). Specifically, the combination therapy resulted in a mean increase in HDL-C of 18.3% and a mean reduction in TG levels of 44.7%.
Chile (23)	*APOA5* Q97X (nonsense mutation p.Gln97Ter)	Not specified	Genome-wide linkage study (via homozygosity mapping strategy) 19 subjects from consanguineous family	Subjects 11 and 12, who had severe HTG, were treated with combinations of fibrates and nicotinic acid, and fibrates with omega-3 fatty acids, subject 13 received atorvastatin	The *APOA5* Q97X mutation was associated with severe HTG and showed no response to statin therapy. Subject 13, who was obese (BMI = 30.8) and treated with atorvastatin, exhibited an intermediate phenotype, with TG levels exceeding 5.6 mmol/L but remaining below 22 mmol/L (borderline high).
Hong Kong (24)	*APOA5* rs662799 (c.-1131T>C)	0.316	Cross-sectional study 386 Chinese patients with HPL: 220 subjects with primary HPL; 166 subjects with FH (showed good compliance with rosuvastatin)	Rosuvastatin (10 mg/day)	The *APOA5* rs662799 was significantly associated with higher baseline TG levels and lower baseline HDL-C levels but showed no association with baseline LDL-C levels. Furthermore, lipid responses to rosuvastatin did not differ across *APOA5* rs662799 genotype groups, irrespective if familial hypercholesterolaemia status or the presence of metabolic syndrome.
Czech (25)	*APOA5* rs662799 (c.-1131T>C), *APOA5* rs3135056 c.56C>G) and *APOA5* rs3135507 (c.457G>A)	*APOA5* rs662799 = 0.08; *APOA5* rs3135506 = 0.10	Case-control study 187 patients with primary dyslipidaemia vs. 470 controls	Low dose (10 mg or 20 mg) of Simvastatin, atorvastatin or lovastatin treatment	Cholesterol reduction was not significantly associated with the type or dose of statin administered. However, individuals with the *APOA5* rs662799 TT genotype (*n* = 154) showed a more favourable response to statin therapy compared with carriers of minor C allele (*n* = 33).
Brazil (26)	*APOA5* rs662799, *APOA5* rs3135506	*APOA5* rs662799 = 0.08, *APOA5* rs3135506 = 0.10	Cross-sectional study 614 HIV patients on stable antiretroviral therapy	# The specific type of lipid-lowering medication (17%) used was not identified in the patients	Carriers of the minor C allele of rs662799 exhibited higher TG levels and lower HDL-C levels compared with individuals with the TT genotype. In contrast, no significant association with LDL-C levels was observed among carriers of the G allele or individuals with the CC genotype of rs3135506.
USA (27)	*APOA5* rs662799, *APOA5* rs3135506 and *APOA5* rs964184	Not specified	Observational study 5,124 adult and children (at least 16 years old) from the original Framingham Study	# FA, Bexarotene	The interaction between the *APOA5* gene and environmental factors, including adiposity, diet, and alcohol consumption, may be attributed to the quantile-dependent expressivity of TG levels. Notably, the genetic effect appears to be more pronounced before the initiation of pharmacological treatment.
China (28)	*APOA5* rs662799	0.33	Case-control and meta-analysis study 210 CHD patients vs. 223 healthy controls	# The specific type of lipid-lowering medication used was not identified in the patients	Smoking and alcohol consumption, as well as the presence of hypertension and diabetes mellitus, were more prevalent among cases than in the control group. Additionally, BMI, TG levels and fasting blood glucose were significantly higher in cases compared with controls (*P* < 0.05). Meta-analysis findings further demonstrated a strong association between the rs662799 polymorphism and an increased risk of CHD (*P* < 0.05).
China (29) (Uighur population of Xinjiang)	*APOA5* rs662799	0.267	Case-control study 408 patients with ischaemic stroke vs. 347 healthy controls	Atorvastatin	The study identified an association between the rs662799 polymorphism and lipid metabolism in individuals with ischaemic stroke. Furthermore, combined genetic analysis revealed that interactions among *LDLR* rs688, *APOA5* rs662799, and *CETP* rs708272 significantly contribute to the susceptibility of ischaemic stroke.
Korea (30)	rs3398424 and rs3135057	Not specified	Prospective study Healthy participants (*n* = 35)	Atorvastatin (80 mg)	Patient age (*P* = 0.003) and genetic polymorphisms, particularly *APOA5* rs3398424 as well as variants in *ABCB1, ABCG2, CETP*, and *CYP7A1, were* associated with increased atorvastatin exposure, reflected by higher AUC and C_max_ values (approximately two-fold higher compared with Caucasians). The atorvastatin AUC_0–24 h_ prediction model was developed based on age and eight genetic variants using multivariate linear regression (adjusted R^2^ = 0.878, *P* < 0.0001).
Malaysia (31)	rs662799	0.450	Cross-sectional retrospective study 229 patients with hyperlipidaemia	Most participants were treated with atorvastatin or simvastatin at low- to moderate-dose regimen (10 to 40 mg/day)	At baseline, *APOA5* rs662799 correlated with higher HDL-C levels (*P* = 0.007). Following statin treatment, the SNP demonstrated improved lipid profiles characterised by elevated HDL-C (*P* = 0.031) and reduced TG levels (*P* = 0.037). However, the *APOA5* rs662799 variant did not influence statin-induced LDL-c lowering effects.

* rs964184 in the *ZPR1* gene was included in this review due to its proximity to the *APOA1-C3-A4-A5* gene cluster on chromosome 11;

# The participants in the study were treated with lipid-lowering drugs, however the specific types of statin used were not reported;

APOA5 = apolipoprotein A5; CAD = coronary artery disease; FA = fenofibric acid; HTG = hypertriglyceridaemia; HPL = hyperlipidaemia; FH = familial hypercholesterolaemia; CHD = coronary heart disease; TG = triglycerides; HDL-C = high-density lipoprotein cholesterol; *CETP* = cholesteryl ester transfer protein; BMI = body mass index; LDL-C = low-density lipoprotein cholesterol; LDLR = low-density lipoprotein receptor; *ABCB1* = ATP-binding cassette sub-family B member 1; *ABCG2* = ATP-binding cassette sub-family G member 2; *CYP7A1* = cytochrome P450 7A1; SNP = single nucleotide polymorphism; MAF = minor allele frequency

## References

[1] Mahley RW, Innerarity TL, Rall SC, Weisgraber KH (1984). Plasma lipoproteins: apolipoprotein structure and function. J Lipid Res.

[2] O’Brien PJ, Alborn WE, Sloan JH, Ulmer M, Boodhoo A, Knierman MD (2005). The novel apolipoprotein A5 is present in human serum, is associated with VLDL, HDL, and chylomicrons, and circulates at very low concentrations compared with other apolipoproteins. Clin Chem.

[3] Charlton Menys V, Durrington PN (2005). Apolipoprotein A5 and hypertriglyceridemia. Clin Chem.

[4] Pennacchio LA, Olivier M, Hubacek JA, Cohen JC, Cox DR, Fruchart JC (2001). An apolipoprotein influencing triglycerides in humans and mice revealed by comparative sequencing. Science.

[5] Garelnabi M, Lor K, Jin J, Chai F, Santanam N (2013). The paradox of ApoA5 modulation of triglycerides: evidence from clinical and basic research. Clin Biochem.

[6] Wong K, Ryan RO (2007). Characterization of apolipoprotein A-V structure and mode of plasma triacylglycerol regulation. Curr Opin Lipidol.

[7] Hegele RA (2009). Plasma lipoproteins: genetic influences and clinical implications. Nat Rev Genet.

[8] Han Y, Dorajoo R, Chang X, Wang L, Khor CC, Sim X (2017). Genome-wide association study identifies a missense variant at *APOA5* for coronary artery disease in multi-ethnic cohorts from Southeast Asia. Sci Rep.

[9] Pennacchio LA, Rubin EM (2003). Apolipoprotein A5, a newly identified gene that affects plasma triglyceride levels in humans and mice. Arterioscler Thromb Vasc Biol.

[10] van de Woestijne AP, Monajemi H, Kalkhoven E, Visseren FLJ (2011). Adipose tissue dysfunction and hypertriglyceridemia: mechanisms and management. Obes Rev.

[11] Gotto AM (1998). Triglyceride as a risk factor for coronary artery disease. Am J Cardiol.

[12] Pejic RN, Lee DT (2006). Hypertriglyceridemia. J Am Board Fam Med.

[13] Vaessen SFC, Schaap FG, Kuivenhoven JA, Groen AK, Hutten BA, Boekholdt SM (2006). Apolipoprotein A-V, triglycerides and risk of coronary artery disease: the prospective EPIC-Norfolk population study. J Lipid Res.

[14] Morjane I, Charoute H, Ouatou S, Elkhattabi L, Benrahma H, Saile R (2020). Association of c.56C>G (rs3135506) apolipoprotein A5 gene polymorphism with coronary artery disease in Moroccan subjects: a case-control study and updated meta-analysis. Cardiol Res Pract.

[15] Hubacek JA, Skodová Z, Lánská V, Adámková V (2008). Apolipoprotein A-V variant (T-1131>C) affects plasma levels of non-high-density lipoprotein cholesterol in Caucasians. Exp Clin Cardiol.

[16] Arksey H, O’Malley L (2005). Scoping studies: towards a methodological framework. Int J Soc Res Methodol.

[17] Moher D, Shamseer L, Clarke M, Ghersi D, Liberati A, Petticrew M (2015). Preferred reporting items for systematic review and meta-analysis protocols (PRISMA-P) 2015 statement. Syst Rev.

[18] Brautbar A, Barbalic M, Chen F, Belmont J, Virani SS, Scherer S (2013). Rare APOA5 promoter variants associated with paradoxical HDL cholesterol decrease in response to fenofibric acid therapy. J Lipid Res.

[19] Clifford AJ, Rincon G, Owens JE, Medrano JF, Moshfegh AJ, Baer DJ (2013). Single nucleotide polymorphisms in *CETP*, *SLC46A1*, *SLC19A1*, *CD36*, *BCMO1*, *APOA5*, and *ABCA1* are significant predictors of plasma HDL in healthy adults. Lipids Health Dis.

[20] van de Woestijne AP, van der Graaf Y, de Bakker PIW, Asselbergs FW, Spiering W, Visseren FLJ (2014). Rs964184 (APOA5-A4-C3-A1) is related to elevated plasma triglyceride levels, but not to an increased risk for vascular events in patients with clinically manifest vascular disease. PLoS One.

[21] Bogari NM, Aljohani A, Amin AA, Al Allaf FA, Dannoun A, Taher MM (2019). A genetic variant c.553G > T (rs2075291) in the apolipoprotein A5 gene is associated with altered triglycerides levels in coronary artery disease (CAD) patients with lipid lowering drug. BMC Cardiovasc Disord.

[22] Brautbar A, Covarrubias D, Belmont J, Lara-Garduno F, Virani SS, Jones PH (2011). Variants in the *APOA5* gene region and the response to combination therapy with statins and fenofibric acid in a randomized clinical trial of individuals with mixed dyslipidemia. Atherosclerosis.

[23] Dussaillant C, Serrano V, Maiz A, Eyheramendy S, Cataldo LR, Chavez M (2012). APOA5 Q97X mutation identified through homozygosity mapping causes severe hypertriglyceridemia in a Chilean consanguineous family. BMC Med Genet.

[24] Hu M, Mak VWL, Tomlinson B (2012). Polymorphisms in apolipoprotein E and apolipoprotein A-V do not influence the lipid response to rosuvastatin but are associated with baseline lipid levels in Chinese patients with hyperlipidemia. J Clin Lipidol.

[25] Hubacek JA, Adamkova V, Prusikova M, Snejdrlova M, Hirschfeldova K, Lanska V (2009). Impact of apolipoprotein A5 variants on statin treatment efficacy. Pharmacogenomics.

[26] Lazzaretti RK, Gasparotto AS, de M, Sassi MG, Polanczyk CA, Kuhmmer R, Silveira JM (2013). Genetic markers associated to dyslipidemia in HIV-infected individuals on HAART. Sci World J.

[27] Williams PT (2020). Gene-environment interactions due to quantile-specific heritability of triglyceride and VLDL concentrations. Sci Rep.

[28] Yang G, Lei MM, Yu CL, Liu XX, An Z, Song CL (2015). Apolipoprotein A5 and apolipoprotein C3 single nucleotide polymorphisms are correlated with an increased risk of coronary heart disease: a case-control and meta-analysis study. Lipids Health Dis.

[29] Yue Y, Liu L, Hu L, Li Y, Mao J, Yang X (2017). The association of lipid metabolism relative gene polymorphisms and ischemic stroke in Han and Uighur population of Xinjiang. Lipids Health Dis.

[30] Kim S, Seo JD, Yun YM, Kim H, Kim TE, Lee T (2022). Pharmacokinetics and genetic factors of atorvastatin in healthy Korean subjects. Front Genet.

[31] Shamsudin AF, Sulong S, Ahmad I, Bakar NS (2024). Association between genetic polymorphisms and other attributing factors with lipid profiles among statin users: a cross-sectional retrospective study. Egypt J Med Hum Genet.

[32] Chang CK, Lin XR, Lin YL, Fang WH, Lin SW, Chang SY (2018). Magnolol-mediated regulation of plasma triglyceride through affecting lipoprotein lipase activity in apolipoprotein A5 knock-in mice. PLoS One.

[33] Theodoropoulos P, Fanaropoulou NM, Manessis A (2024). Unmasking a rare genetic mutation: the importance of genetic testing in refractory hypertriglyceridemia. AACE Clin Case Rep.

[34] Triglyceride Coronary Disease Genetics Consortium and Emerging Risk Factors Collaboration (2010). Triglyceride-mediated pathways and coronary disease: collaborative analysis of 101 studies. Lancet.

[35] Tsutsumi K (2003). Lipoprotein lipase and atherosclerosis. Curr Vasc Pharmacol.

[36] San Mauro Martín I, Blumenfeld Olivares JA, Pérez Arruche E, Arce Delgado E, Ciudad Cabañas MJ, Garicano Vilar E (2018). Genomic influence in the prevention of cardiovascular diseases with a sterol-based treatment. Diseases.

[37] Guardiola M, Ferré R, Salazar J, Alonso-Villaverde C, Coll B, Parra S (2006). Protease inhibitor-associated dyslipidemia in HIV-infected patients is strongly influenced by the APOA5 -1131T->C gene variation. Clin Chem.

[38] Hodoğlugil U, Tanyolaç S, Williamson DW, Huang Y, Mahley RW (2006). Apolipoprotein A-V: a potential modulator of plasma triglyceride levels in Turks. J Lipid Res.

[39] de Andrade FM, Maluf SW, Schuch JB, Voigt F, Barros AC, Lucatelli JF (2011). The influence of the S19W SNP of the APOA5 gene on triglyceride levels in southern Brazil: interactions with the APOE gene, sex and menopause status. Nutr Metab Cardiovasc Dis.

[40] Yuan G, Al-Shali KZ, Hegele RA (2007). Hypertriglyceridemia: its etiology, effects and treatment. CMAJ.

[41] de Almeida ERD, Reiche EMV, Kallaur AP, Flauzino T, Watanabe MAE (2013). The roles of genetic polymorphisms and human immunodeficiency virus infection in lipid metabolism. Biomed Res Int.

[42] Oliva CP, Carubbi F, Schaap FG, Bertolini S, Calandra S (2008). Hypertriglyceridaemia and low plasma HDL in a patient with apolipoprotein A-V deficiency due to a novel mutation in the *APOA5* gene. J Intern Med.

[43] Klop B, Elte JWF, Cabezas MC (2013). Dyslipidemia in obesity: mechanisms and potential targets. Nutrients.

[44] Hubacek JA (2016). Apolipoprotein A5 fifteen years anniversary: lessons from genetic epidemiology. Gene.

[45] Zainon R, Shamsudin AF, Zulkafli Z, Bakar NS (2023). Association between SORT1/CELSR2/PSRC1 rs646776 polymorphism and statin-affected plasma lipid levels. Biomed Res Ther.

[46] Young SG, Fong LG (2012). Lowering plasma cholesterol by raising LDL receptors—revisited. N Engl J Med.

[47] Ding Y, Zhu MA, Wang ZX, Zhu J, Feng JB, Li DS (2012). Associations of polymorphisms in the apolipoprotein APOA1-C3-A5 gene cluster with acute coronary syndrome. Biomed Res Int.

[48] Do R, Willer CJ, Schmidt EM, Sengupta S, Gao C, Peloso GM (2013). Common variants associated with plasma triglycerides and risk for coronary artery disease. Nat Genet.

[49] Kessler T, Vilne B, Schunkert H (2016). The impact of genome-wide association studies on the pathophysiology and therapy of cardiovascular disease. EMBO Mol Med.

[50] Dallongeville J, Cottel D, Montaye M, Codron V, Amouyel P, Helbecque N (2006). Impact of *APOA5/A4/C*3 genetic polymorphisms on lipid variables and cardiovascular disease risk in French men. Int J Cardiol.

[51] Hubacek J, Škodová Z, Adámková V, Lánská V, Poledne R (2004). The influence of *APOAV* polymorphisms (T-1131>C and S19>W) on plasma triglyceride levels and risk of myocardial infarction. Clin Genet.

[52] Abedi AH, Yıldırım Şimşir I, Bayram F, Onay H, Özgür S, Mcintyre A (2023). Genetic variants associated with severe hypertriglyceridemia: LPL, APOC2, APOA5, GPIHBP1, LMF1, and APOE. Turk Kardiyol Dern Ars.

[53] Grallert H, Sedlmeier EM, Huth C, Kolz M, Heid IM, ogaringer C (2007). *APOA5* variants and metabolic syndrome in Caucasians. J Lipid Res.

[54] Pi Y, Zhang L, Yang Q, Li B, Guo L, Fang C (2012). Apolipoprotein A5 gene promoter region-*1131T/C* polymorphism is associated with risk of ischemic stroke and elevated triglyceride levels: a meta-analysis. Cerebrovasc Dis.

[55] Bakker-Arkema RG, Davidson MH, Goldstein RJ, Davignon J, Isaacsohn JL, Weiss SR (1996). Efficacy and safety of a new HMG-CoA reductase inhibitor, atorvastatin, in patients with hypertriglyceridemia. JAMA.

[56] Shamsudin AF, Bakar NS (2023). Gender differences in the association between cholesteryl esters transfer protein polymorphism (rs708272) and plasma lipid levels in hyperlipidaemic participants at Hospital Universiti Sains Malaysia. Malays J Med Sci.

[57] Guyton JR (2004). Extended-release niacin for modifying the lipoprotein profile. Expert Opin Pharmacother.

[58] The HPS2-THRIVE Collaborative Group (2014). Effects of extended-release niacin with laropiprant in high-risk patients. N Engl J Med.

[59] Dumolt JH, Rideout TC (2017). The lipid-lowering effects and associated mechanisms of dietary phytosterol supplementation. Curr Pharm Des.

[60] Abumweis SS, Barake R, Jones PJH (2008). Plant sterols/stanols as cholesterol lowering agents: a meta-analysis of randomized controlled trials. Food Nutr Res.

