# Supplementary material for: Unravelling the Influence of Apolipoprotein A5 Gene Polymorphisms on Lipid Profiles of Statin-Treated Patients: A Scoping Review
Source: Malays J Med Sci. 2026 Jun 30;33(3):11–26. doi: 10.21315/mjms-04-2026-240 (PMC13739298; doi:10.21315/mjms-04-2026-240)

Search from April 2002 – 2020

PUBMED CLINICAL QUERIES

(statin* OR “Hydroxymethylglutaryl-CoA Reductase Inhibitors” OR simvastatin OR atorvastatin OR rosuvastatin OR pravastatin OR lovastatin OR fluvastatin) AND (“APOA5 polymorphism” OR “APOA5 mutant” OR “APOA5 carrier” OR “APOA5 variant” OR “Apolipoprotein A-V” OR “Apolipoprotein A5” OR “Polymorphism, Single Nucleotide”) AND (“cholesterol lower*” OR “cholesterol reduction” OR “cholesterol decrease” OR cholesterol OR LDL OR epicholesterol)

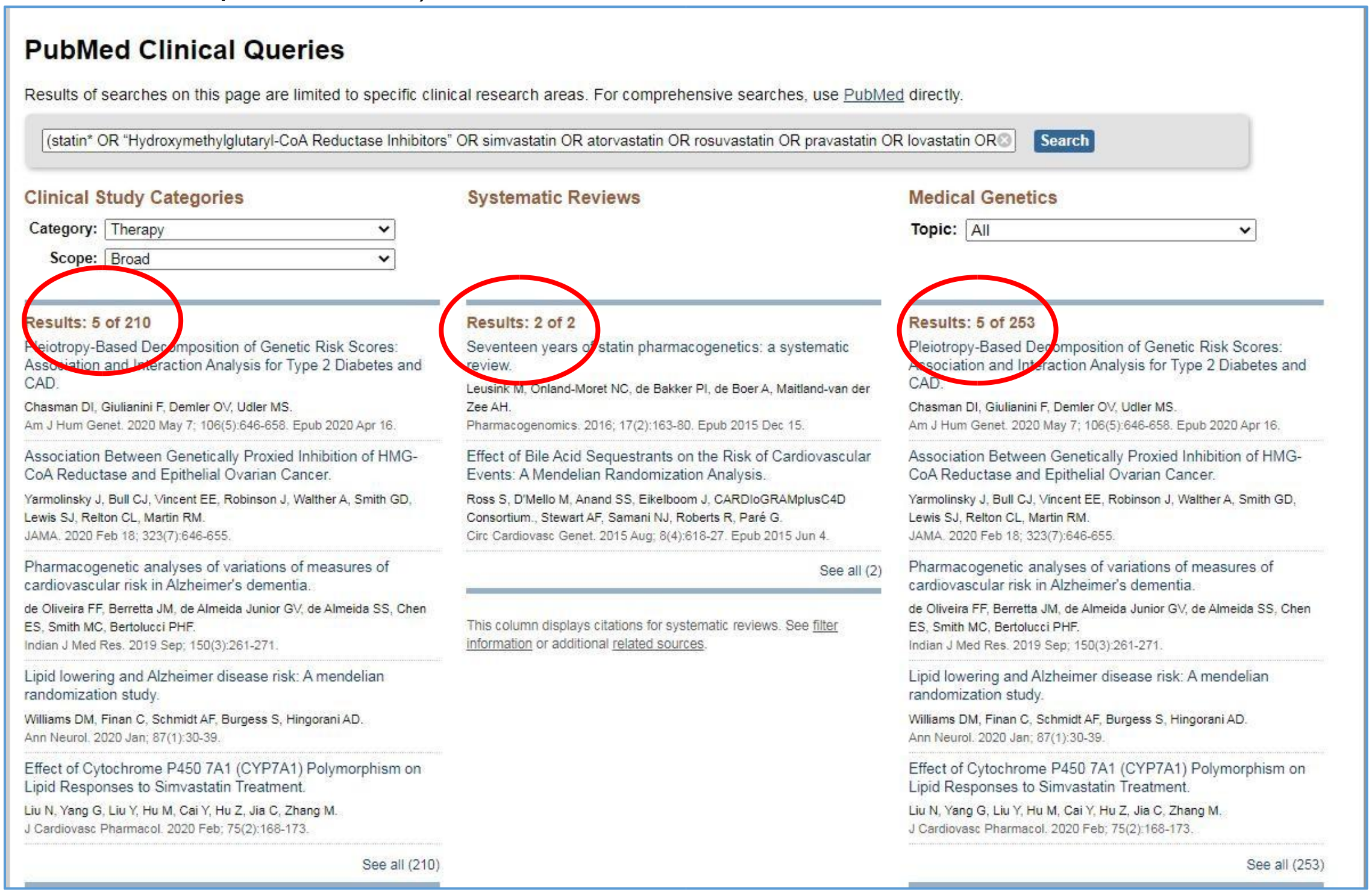

PubMed Clinical Queries

Results of searches on this page are limited to specific clinical research areas. For comprehensive searches, use PubMed directly.

(statin* OR “Hydroxymethylglutaryl-CoA Reductase Inhibitors” OR simvastatin OR atorvastatin OR rosuvastatin OR pravastatin OR lovastatin OR | Search

Clinical Study Categories

Category: Therapy

Scope: Broad

Results: 5 of 210

Pleiotropy-Based Decomposition of Genetic Risk Scores: Association and Interaction Analysis for Type 2 Diabetes and CAD.
Chasman DI, Giulianini F, Demler OV, Udler MS.
Am J Hum Genet. 2020 May 7; 106(5):646-658. Epub 2020 Apr 16.

Association Between Genetically Proxied Inhibition of HMG-CoA Reductase and Epithelial Ovarian Cancer.
Yarmolinsky J, Bull CJ, Vincent EE, Robinson J, Walther A, Smith GD, Lewis SJ, Relton CL, Martin RM.
JAMA. 2020 Feb 18; 323(7):646-655.

Pharmacogenetic analyses of variations of measures of cardiovascular risk in Alzheimer's dementia.
de Oliveira FF, Berretta JM, de Almeida Junior GV, de Almeida SS, Chen ES, Smith MC, Bertolucci PHF.
Indian J Med Res. 2019 Sep; 150(3):261-271.

Lipid lowering and Alzheimer disease risk: A mendelian randomization study.
Williams DM, Finan C, Schmidt AF, Burgess S, Hingorani AD.
Ann Neurol. 2020 Jan; 87(1):30-39.

Effect of Cytochrome P450 7A1 (CYP7A1) Polymorphism on Lipid Responses to Simvastatin Treatment.
Liu N, Yang G, Liu Y, Hu M, Cai Y, Hu Z, Jia C, Zhang M.
J Cardiovasc Pharmacol. 2020 Feb; 75(2):168-173.

See all (210)

Systematic Reviews

Results: 2 of 2

Seventeen years of statin pharmacogenetics: a systematic review.
Leusink M, Onland-Moret NC, de Bakker PI, de Boer A, Maitland-van der Zee AH.
Pharmacogenomics. 2016; 17(2):163-80. Epub 2015 Dec 15.

Effect of Bile Acid Sequestrants on the Risk of Cardiovascular Events: A Mendelian Randomization Analysis.
Ross S, D'Mello M, Anand SS, Eikelboom J, CARDIoGRAMplusC4D Consortium., Stewart AF, Samani NJ, Roberts R, Paré G.
Circ Cardiovasc Genet. 2015 Aug; 8(4):618-27. Epub 2015 Jun 4.

See all (2)

This column displays citations for systematic reviews. See filter information or additional related sources.

Medical Genetics

Topic: All

Results: 5 of 253

Pleiotropy-Based Decomposition of Genetic Risk Scores: Association and Interaction Analysis for Type 2 Diabetes and CAD.
Chasman DI, Giulianini F, Demler OV, Udler MS.
Am J Hum Genet. 2020 May 7; 106(5):646-658. Epub 2020 Apr 16.

Association Between Genetically Proxied Inhibition of HMG-CoA Reductase and Epithelial Ovarian Cancer.
Yarmolinsky J, Bull CJ, Vincent EE, Robinson J, Walther A, Smith GD, Lewis SJ, Relton CL, Martin RM.
JAMA. 2020 Feb 18; 323(7):646-655.

Pharmacogenetic analyses of variations of measures of cardiovascular risk in Alzheimer's dementia.
de Oliveira FF, Berretta JM, de Almeida Junior GV, de Almeida SS, Chen ES, Smith MC, Bertolucci PHF.
Indian J Med Res. 2019 Sep; 150(3):261-271.

Lipid lowering and Alzheimer disease risk: A mendelian randomization study.
Williams DM, Finan C, Schmidt AF, Burgess S, Hingorani AD.
Ann Neurol. 2020 Jan; 87(1):30-39.

Effect of Cytochrome P450 7A1 (CYP7A1) Polymorphism on Lipid Responses to Simvastatin Treatment.
Liu N, Yang G, Liu Y, Hu M, Cai Y, Hu Z, Jia C, Zhang M.
J Cardiovasc Pharmacol. 2020 Feb; 75(2):168-173.

See all (253)

History and Search Details

Download | Delete

Search	Actions	Details	Query	Results	Time
#3	•••	! >	Search: **(Medical Genetics[filter]) AND ((statin* OR "Hydroxymethylglutaryl-CoA Reductase Inhibitors" OR simvastatin OR atorvastatin OR rosuvastatin OR pravastatin OR lovastatin OR fluvastatin) AND ("APOA5 polymorphism" OR "APOA5 mutant" OR "APOA5 carrier" OR "APOA5 variant" OR "Apolipoprotein A-V" OR "Apolipoprotein A5" OR "Polymorphism, Single Nucleotide") AND ("cholesterol lower*" OR "cholesterol reduction" OR "cholesterol decrease" OR cholesterol OR LDL OR epicholesterol))**	254	03:33:20
#2	•••	! >	Search: **systematic[sb] AND ((statin* OR "Hydroxymethylglutaryl-CoA Reductase Inhibitors" OR simvastatin OR atorvastatin OR rosuvastatin OR pravastatin OR lovastatin OR fluvastatin) AND ("APOA5 polymorphism" OR "APOA5 mutant" OR "APOA5 carrier" OR "APOA5 variant" OR "Apolipoprotein A-V" OR "Apolipoprotein A5" OR "Polymorphism, Single Nucleotide") AND ("cholesterol lower*" OR "cholesterol reduction" OR "cholesterol decrease" OR cholesterol OR LDL OR epicholesterol))**	2	03:31:20
#1	•••	! >	Search: **(Therapy/Broad[filter]) AND ((statin* OR "Hydroxymethylglutaryl-CoA Reductase Inhibitors" OR simvastatin OR atorvastatin OR rosuvastatin OR pravastatin OR lovastatin OR fluvastatin) AND ("APOA5 polymorphism" OR "APOA5 mutant" OR "APOA5 carrier" OR "APOA5 variant" OR "Apolipoprotein A-V" OR "Apolipoprotein A5" OR "Polymorphism, Single Nucleotide") AND ("cholesterol lower*" OR "cholesterol reduction" OR "cholesterol decrease" OR cholesterol OR LDL OR epicholesterol))**	210	03:15:21

PUBMED & MEDLINE

History and Search Details

Download | Delete

Search	Actions	Details	Query	Results	Time
#5	•••	! >	Search: **#1 AND #2 AND #3** Filters: **MEDLINE**	10	03:39:24
#4	•••	! >	Search: **#1 AND #2 AND #3**	10	03:39:19
#3	•••	>	Search: **"cholesterol lower*"[Title/Abstract] OR "cholesterol reduction"[Title/Abstract] OR "cholesterol decrease"[Title/Abstract] OR cholesterol[Title/Abstract] OR LDL[Title/Abstract] OR epicholesterol[Title/Abstract]**	270,487	03:38:27
#2	•••	! >	Search: **"APOA5 polymorphism"[Title/Abstract] OR "APOA5 mutant"[Title/Abstract] OR "APOA5 carrier"[Title/Abstract] OR "APOA5 variant"[Title/Abstract] OR "Apolipoprotein A-V"[Title/Abstract] OR "Apolipoprotein A5"[Title/Abstract] OR "Polymorphism, Single Nucleotide"[Title/Abstract]**	601	03:38:03
#1	•••	>	Search: **statin*[Title/Abstract] OR "Hydroxymethylglutaryl-CoA Reductase Inhibitors"[Title/Abstract] OR simvastatin[Title/Abstract] OR atorvastatin[Title/Abstract] OR rosuvastatin[Title/Abstract] OR pravastatin[Title/Abstract] OR lovastatin[Title/Abstract] OR fluvastatin[Title/Abstract]**	58,994	03:37:31

Showing 1 to 5 of 5 entries

COCHRANE LIBRARY

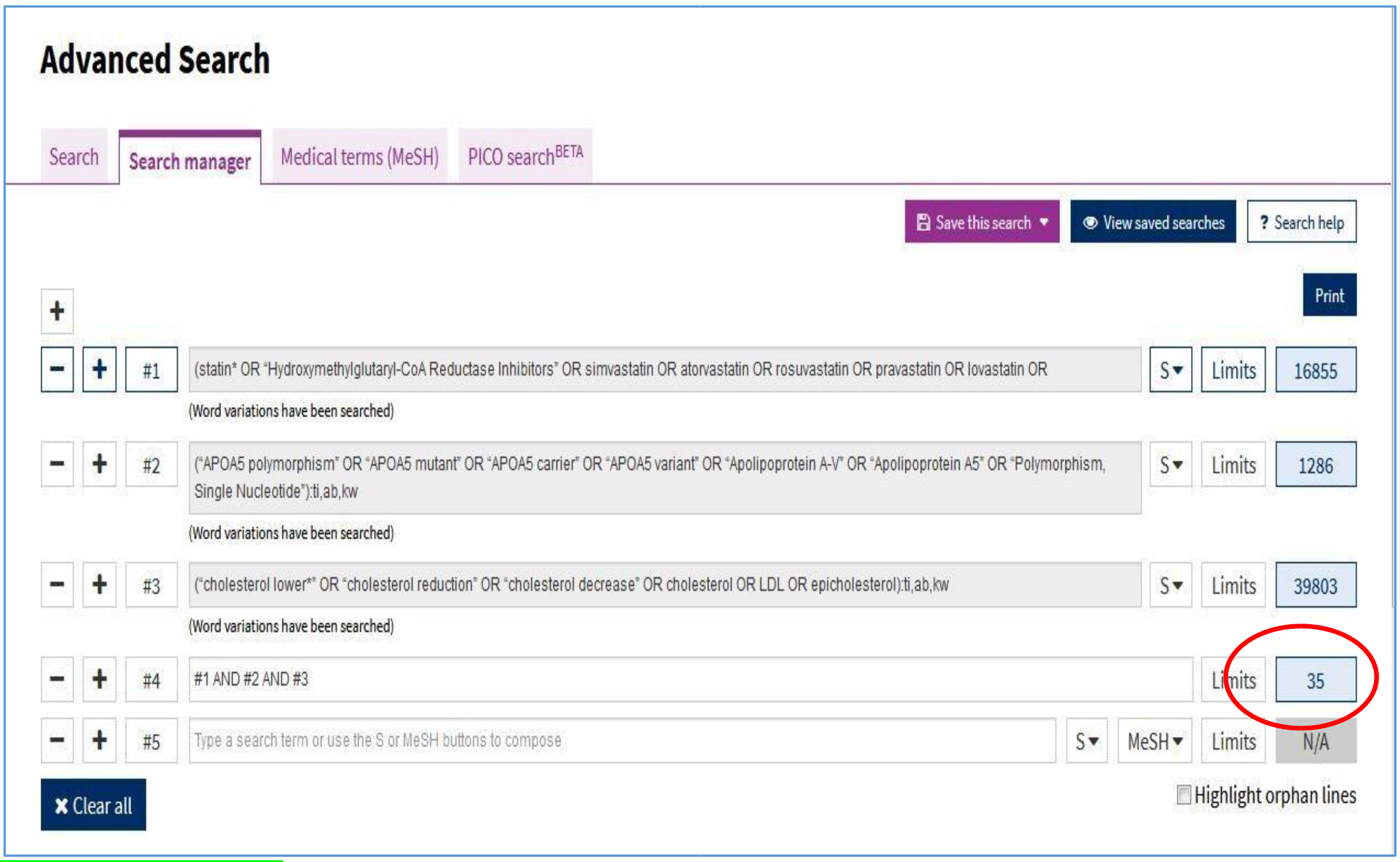

EBSCOHOST-CINAHL

ABSTRACT

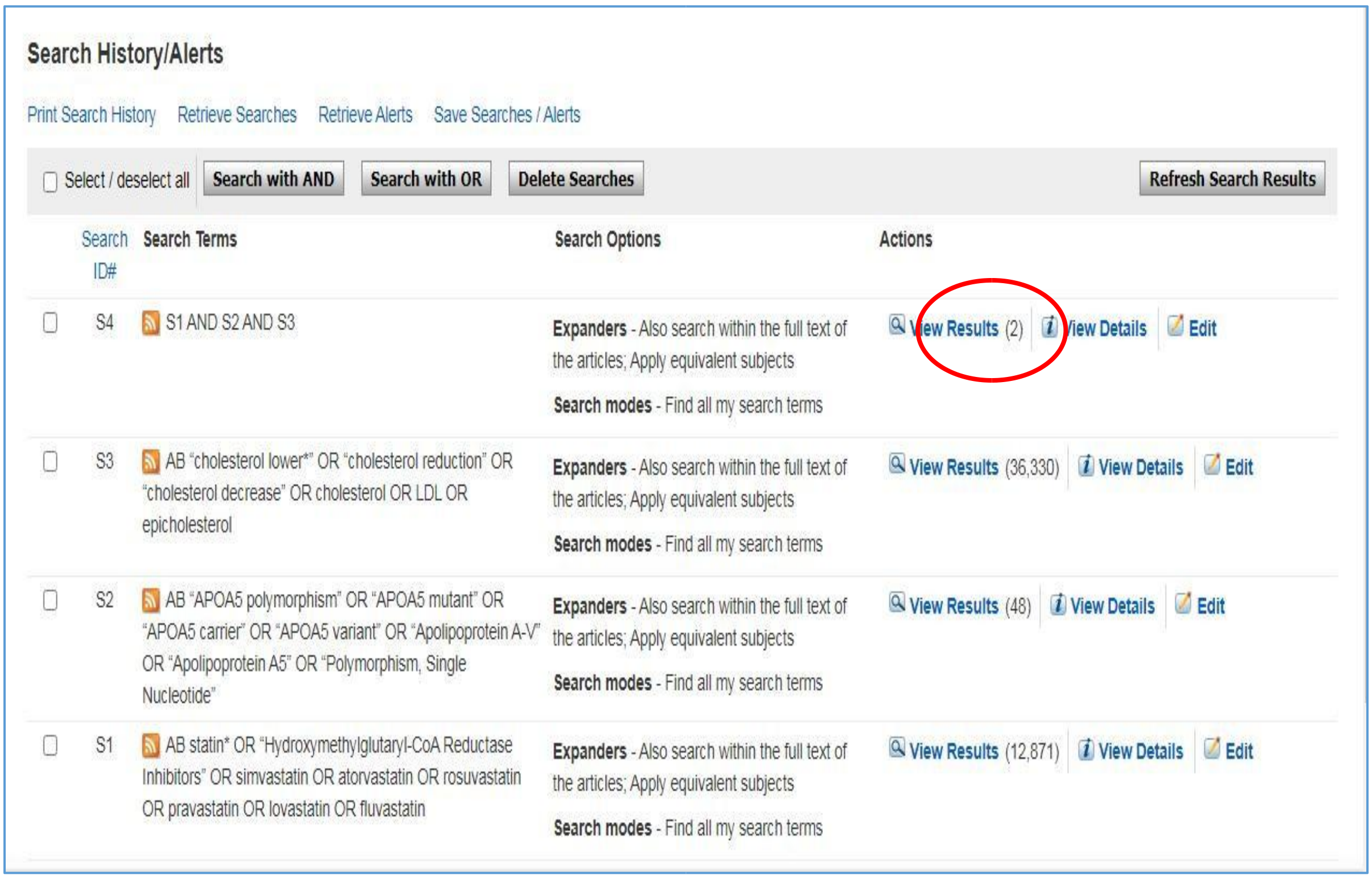

ALL TEXT

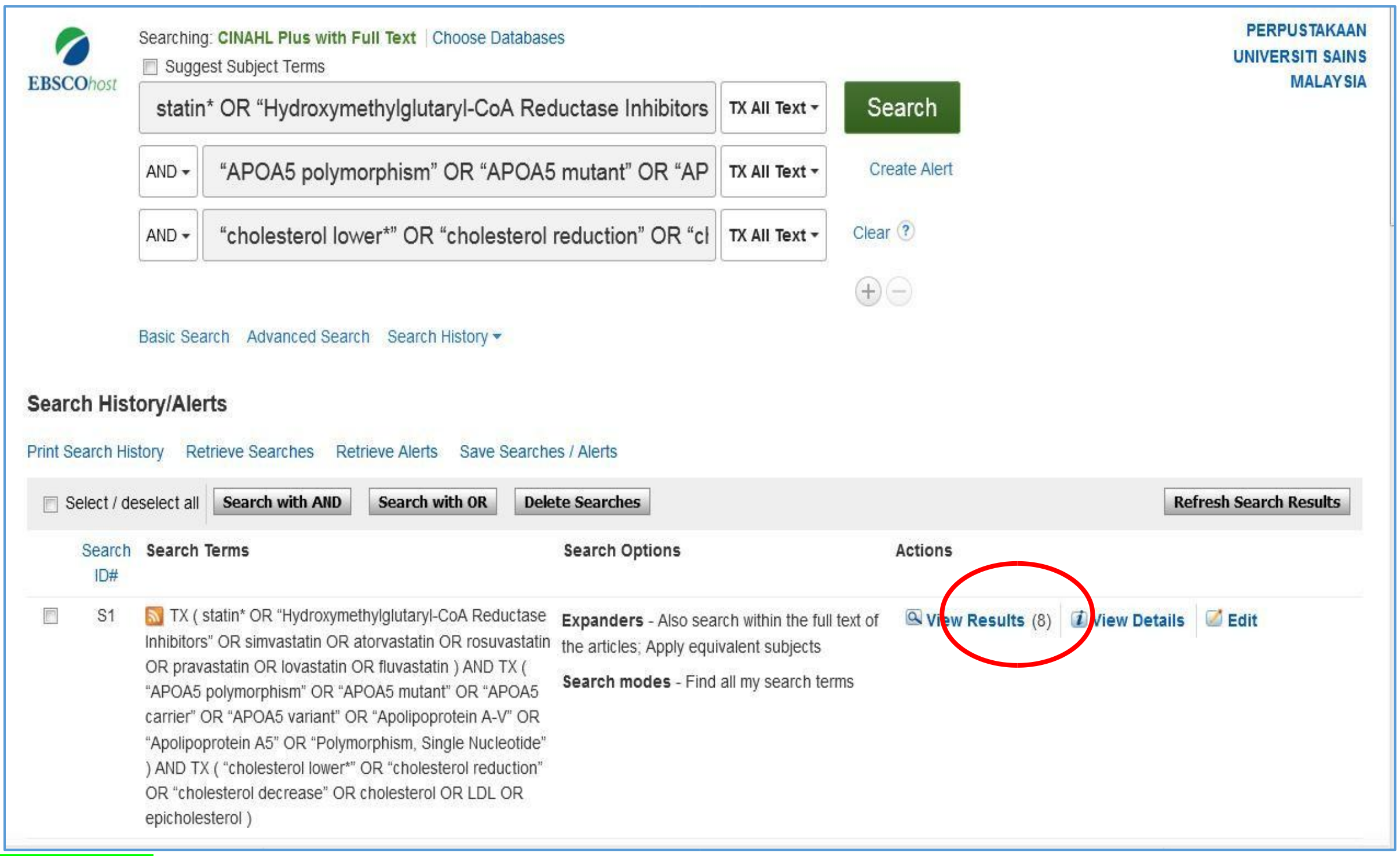

PROQUEST

ABSTRACT

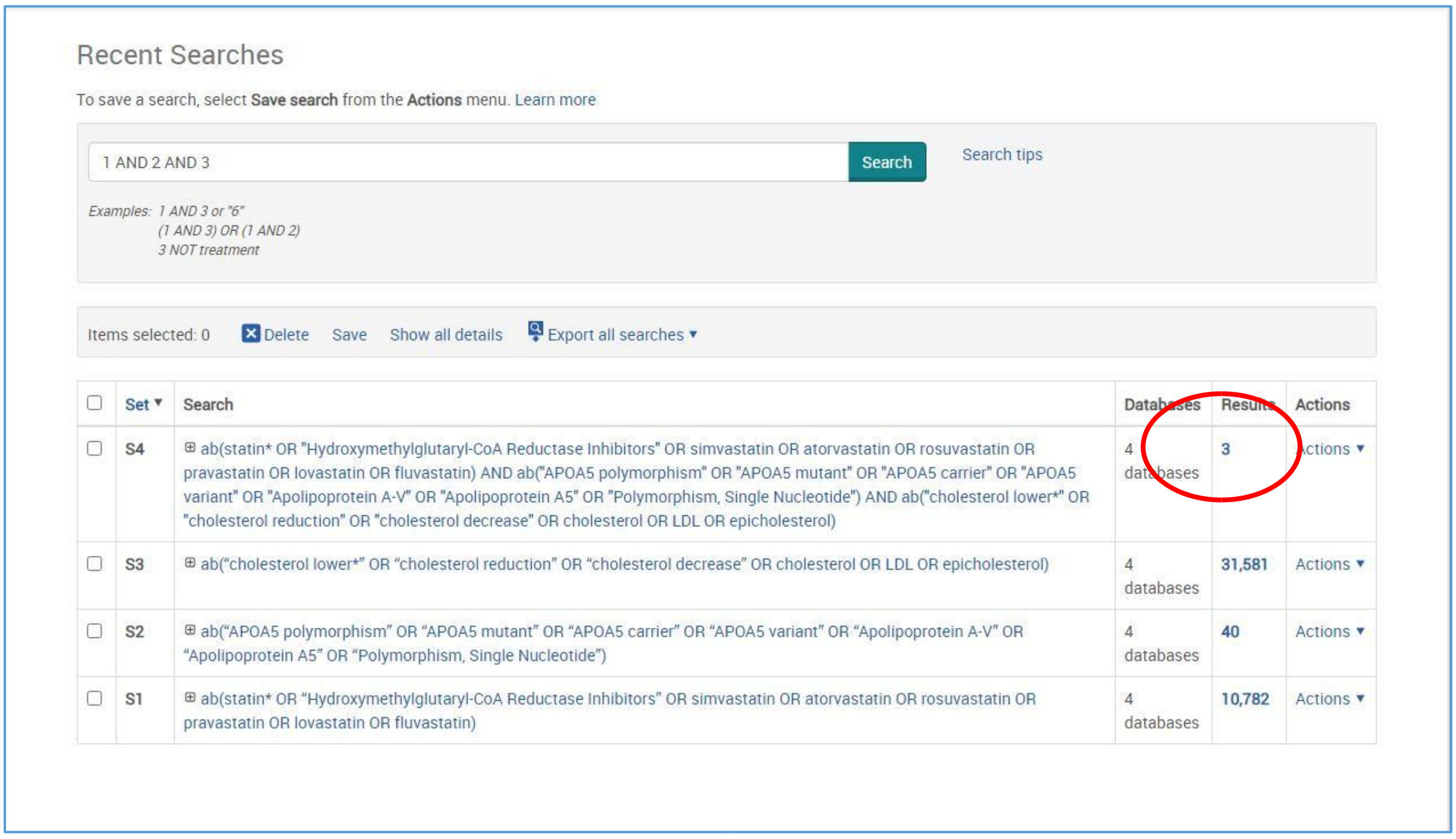

Set	Search	Databases	Results	Actions
S4	ab(statin* OR "Hydroxymethylglutaryl-CoA Reductase Inhibitors" OR simvastatin OR atorvastatin OR rosuvastatin OR pravastatin OR lovastatin OR fluvastatin) AND ab("APOA5 polymorphism" OR "APOA5 mutant" OR "APOA5 carrier" OR "APOA5 variant" OR "Apolipoprotein A-V" OR "Apolipoprotein A5" OR "Polymorphism, Single Nucleotide") AND ab("cholesterol lower*" OR "cholesterol reduction" OR "cholesterol decrease" OR cholesterol OR LDL OR epicholesterol)	4 databases	3	Actions
S3	ab("cholesterol lower*" OR "cholesterol reduction" OR "cholesterol decrease" OR cholesterol OR LDL OR epicholesterol)	4 databases	31,581	Actions
S2	ab("APOA5 polymorphism" OR "APOA5 mutant" OR "APOA5 carrier" OR "APOA5 variant" OR "Apolipoprotein A-V" OR "Apolipoprotein A5" OR "Polymorphism, Single Nucleotide")	4 databases	40	Actions
S1	ab(statin* OR "Hydroxymethylglutaryl-CoA Reductase Inhibitors" OR simvastatin OR atorvastatin OR rosuvastatin OR pravastatin OR lovastatin OR fluvastatin)	4 databases	10,782	Actions

DOCUMENT TEXT

Set ▾	Search	Databases	Results	Actions
S8	⊟ ft(statin* OR "Hydroxymethylglutaryl-CoA Reductase Inhibitors" OR simvastatin OR atorvastatin OR rosuvastatin OR pravastatin OR lovastatin OR fluvastatin) AND ft("APOA5 polymorphism" OR "APOA5 mutant" OR "APOA5 carrier" OR "APOA5 variant" OR "Apolipoprotein A-V" OR "Apolipoprotein A5" OR "Polymorphism, Single Nucleotide") AND ft("cholesterol lower*" OR "cholesterol reduction" OR "cholesterol decrease" OR cholesterol OR LDL OR epicholesterol)<br>Databases: Coronavirus Research Database<br>Ebook Central<br>ProQuest Dissertations & Theses Global<br>Publicly Available Content Database<br>*These databases are searched for part of your query.*	4 databases	166	Actions ▾
S7	⊟ ft("cholesterol lower*" OR "cholesterol reduction" OR "cholesterol decrease" OR cholesterol OR LDL OR epicholesterol) ✔ Limits applied<br>Databases: Coronavirus Research Database<br>Ebook Central<br>ProQuest Dissertations & Theses Global<br>Publicly Available Content Database<br>*These databases are searched for part of your query.*<br>Narrowed by: Full text: Full text	4 databases	224,377	Actions ▾
S6	⊟ ft("APOA5 polymorphism" OR "APOA5 mutant" OR "APOA5 carrier" OR "APOA5 variant" OR "Apolipoprotein A-V" OR "Apolipoprotein A5" OR "Polymorphism, Single Nucleotide") ✔ Limits applied<br>Databases: Coronavirus Research Database<br>Ebook Central<br>ProQuest Dissertations & Theses Global<br>Publicly Available Content Database<br>*These databases are searched for part of your query.*<br>Narrowed by: Full text: Full text	4 databases	653	Actions ▾
S5	⊟ ft(statin* OR "Hydroxymethylglutaryl-CoA Reductase Inhibitors" OR simvastatin OR atorvastatin OR rosuvastatin OR pravastatin OR lovastatin OR fluvastatin) ✔ Limits applied<br>Databases: Coronavirus Research Database<br>Ebook Central<br>ProQuest Dissertations & Theses Global<br>Publicly Available Content Database<br>*These databases are searched for part of your query.*<br>Narrowed by: Full text: Full text	4 databases	629,059	Actions ▾

SCIENCE DIRECT (This database only approved for 8 boolean connectors)

(statin OR “Hydroxymethylglutaryl-CoA Reductase Inhibitors”) AND (“APOA5 polymorphism” OR “Apolipoprotein A-V” OR “Apolipoprotein A5”) AND (“cholesterol lowering” OR “cholesterol reduction” OR “cholesterol decrease”)

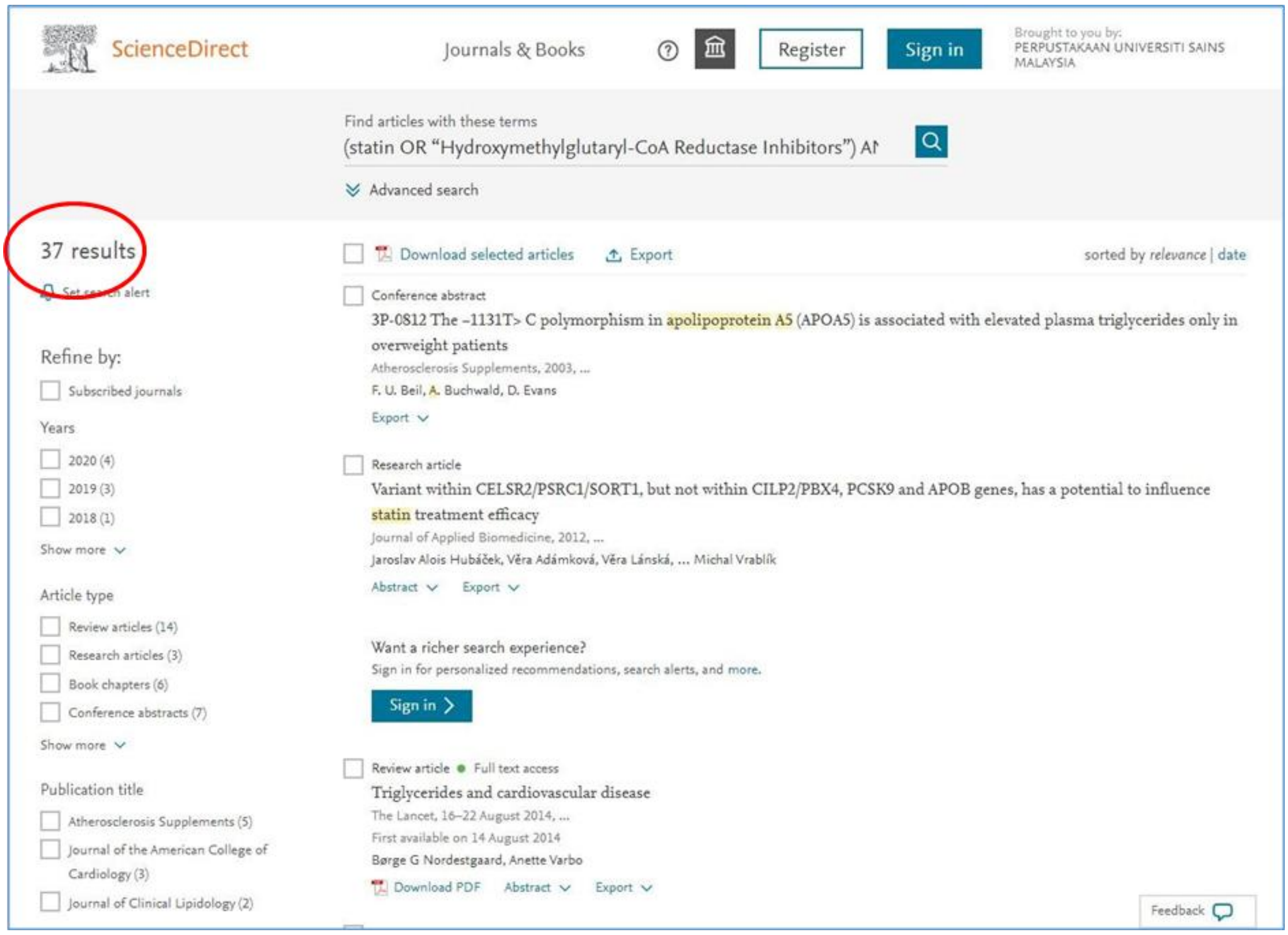

Additional search from 2021 – April 2026

Search Strategy

Element	Term
Patient	statin* OR "Hydroxymethylglutaryl-CoA Reductase Inhibitors" OR simvastatin OR atorvastatin OR rosuvastatin OR pravastatin OR lovastatin OR fluvastatin
Intervention	"APOA5 polymorphism" OR "APOA5 mutant" OR "APOA5 carrier" OR "APOA5 variant" OR "Apolipoprotein A-V" OR "Apolipoprotein A5" OR "Polymorphism, Single Nucleotide"
Comparison	-
Outcome	"cholesterol lower*" OR "cholesterol reduction" OR "cholesterol decrease" OR cholesterol OR "LDL" OR "HDL"

(statin* OR "Hydroxymethylglutaryl-CoA Reductase Inhibitors" OR simvastatin OR atorvastatin OR rosuvastatin OR pravastatin OR lovastatin OR fluvastatin) AND ("APOA5 polymorphism" OR "APOA5 mutant" OR "APOA5 carrier" OR "APOA5 variant" OR "Apolipoprotein A-V" OR "Apolipoprotein A5" OR "Polymorphism, Single Nucleotide") AND ("cholesterol lower*" OR "cholesterol reduction" OR "cholesterol decrease" OR cholesterol OR "LDL" OR "HDL")

PUBMED CLINICAL QUERIES

1) **Result : 85**

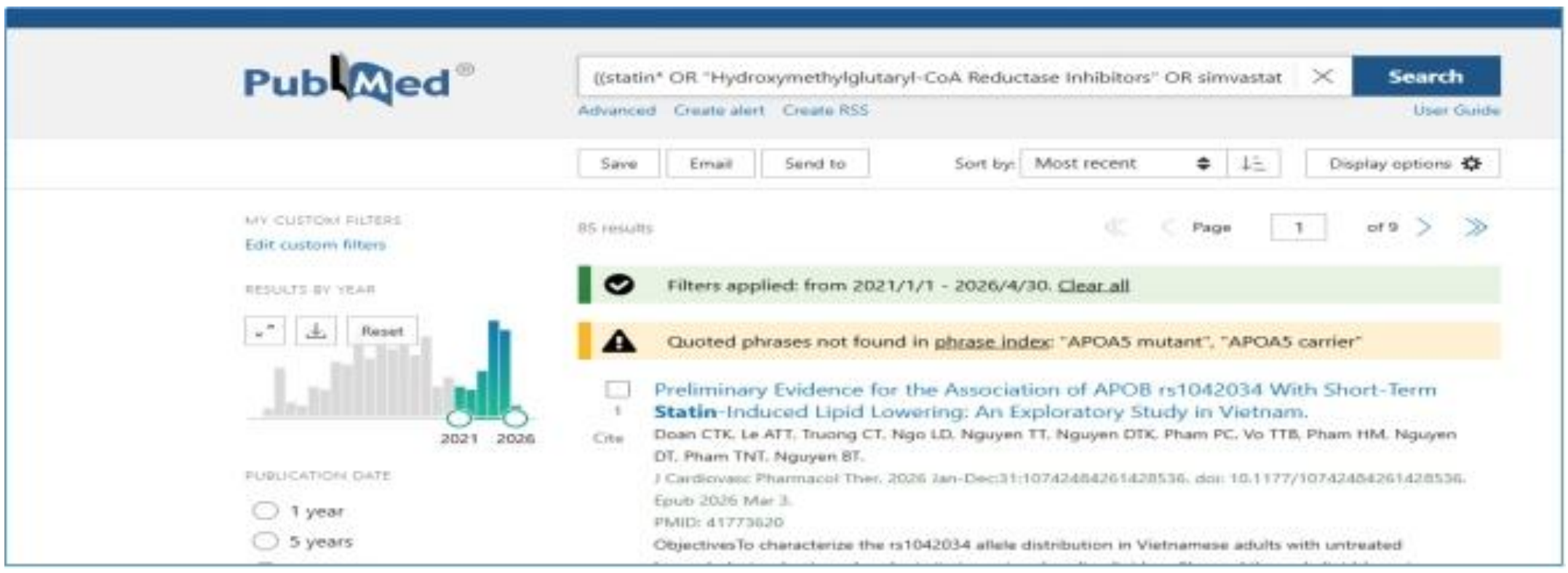

2) **Filter : Review (9 result) –** excluded from the included studies

PUBMED (MEDLINE)

Result : 1

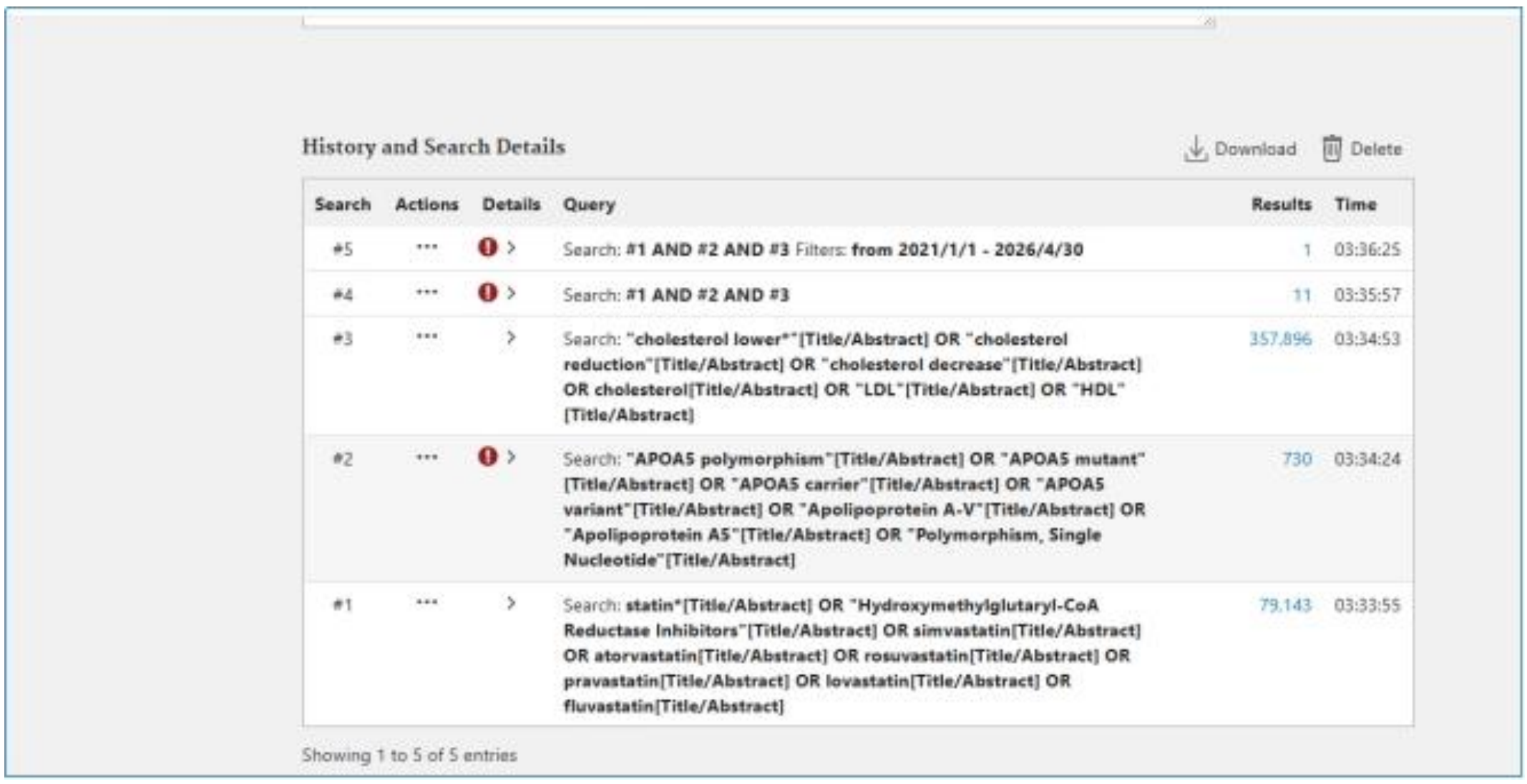

History and Search Details

Search	Actions	Details	Query	Results	Time
#5	•••	! >	Search: #1 AND #2 AND #3 Filters: from 2021/1/1 - 2026/4/30	1	03:36:25
#4	•••	! >	Search: #1 AND #2 AND #3	11	03:35:57
#3	•••	>	Search: "cholesterol lower*"[Title/Abstract] OR "cholesterol reduction"[Title/Abstract] OR "cholesterol decrease"[Title/Abstract] OR cholesterol[Title/Abstract] OR "LDL"[Title/Abstract] OR "HDL"[Title/Abstract]	357,896	03:34:53
#2	•••	! >	Search: "APOA5 polymorphism"[Title/Abstract] OR "APOA5 mutant"[Title/Abstract] OR "APOA5 carrier"[Title/Abstract] OR "APOA5 variant"[Title/Abstract] OR "Apolipoprotein A-V"[Title/Abstract] OR "Apolipoprotein A5"[Title/Abstract] OR "Polymorphism, Single Nucleotide"[Title/Abstract]	730	03:34:24
#1	•••	>	Search: statin*[Title/Abstract] OR "Hydroxymethylglutaryl-CoA Reductase Inhibitors"[Title/Abstract] OR simvastatin[Title/Abstract] OR atorvastatin[Title/Abstract] OR rosuvastatin[Title/Abstract] OR pravastatin[Title/Abstract] OR lovastatin[Title/Abstract] OR fluvastatin[Title/Abstract]	79,143	03:33:55

Showing 1 to 5 of 5 entries

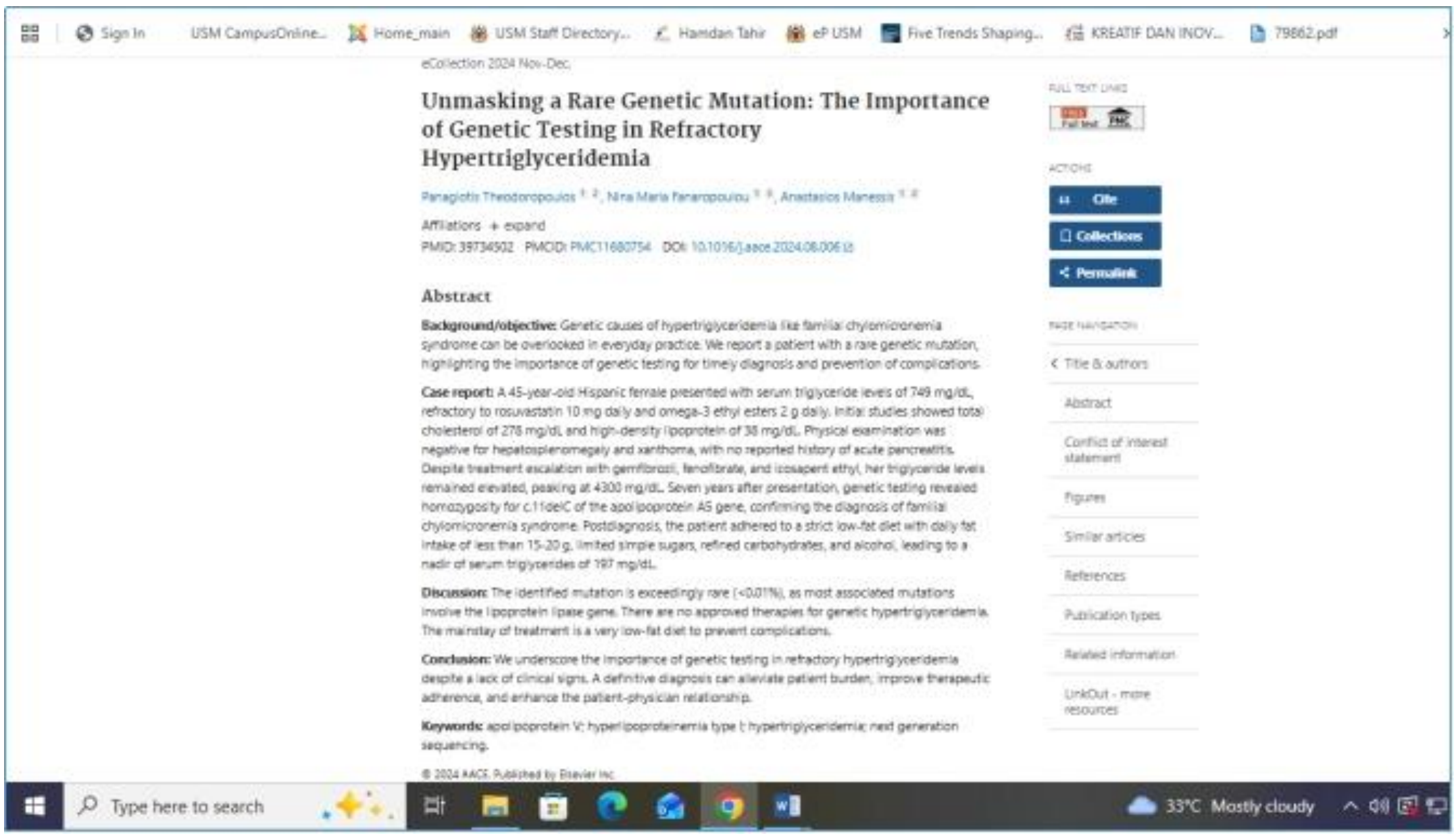

COCHRANE LIBRARY

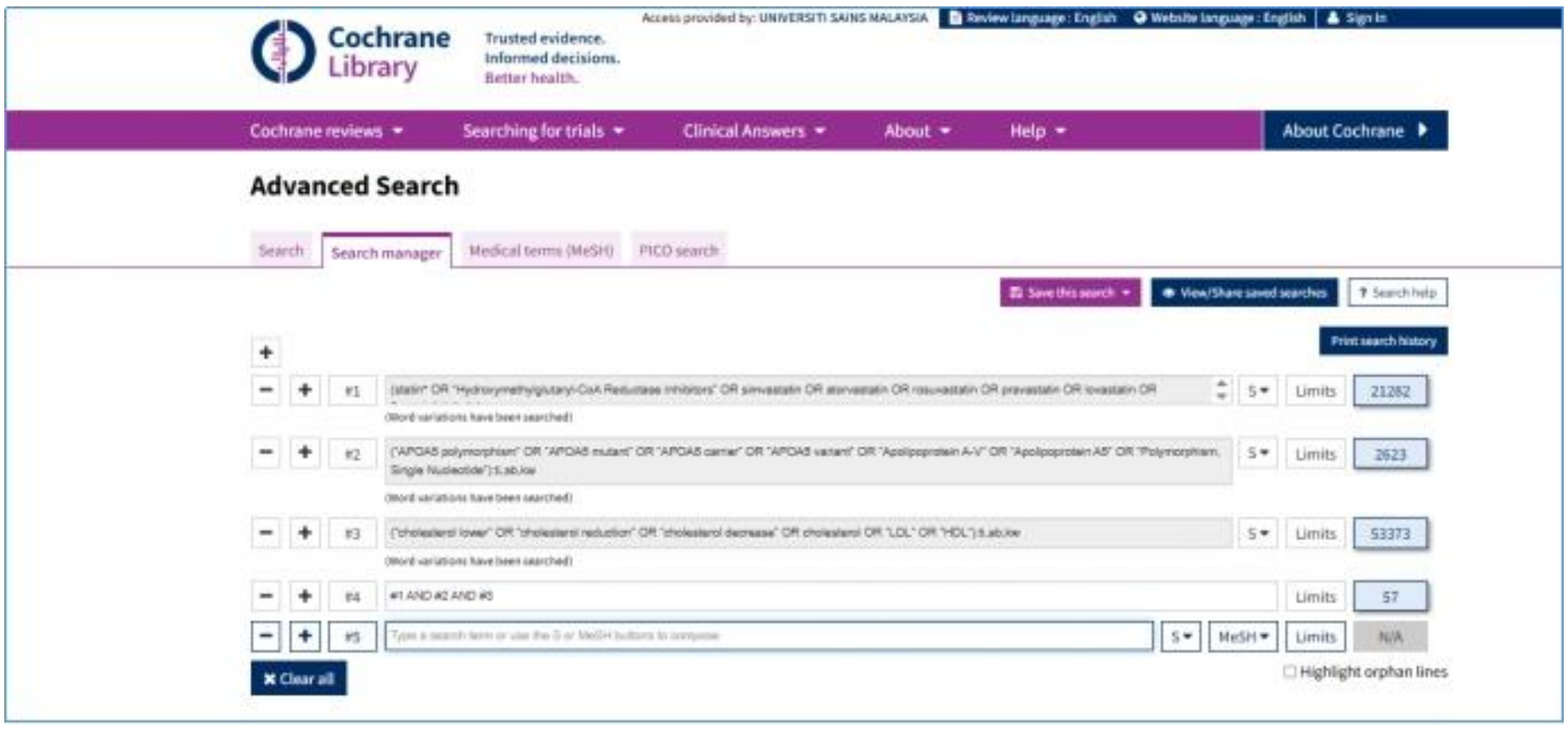

Cochrane (2021 – 2026)

Result : 3 citations (they are excluded from the included studies since they are ongoing clinical trials)

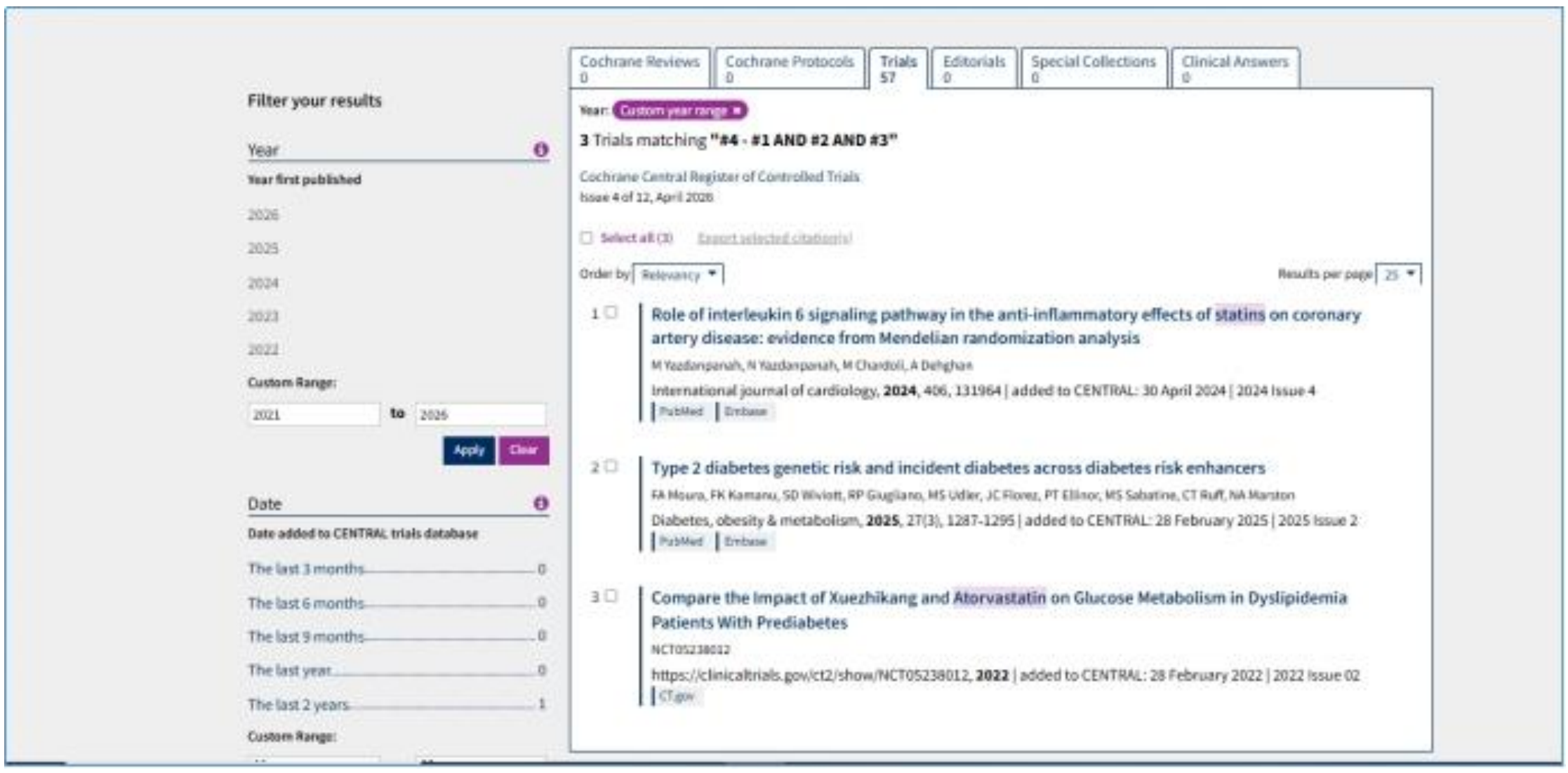

SCIENCE DIRECT –

Result : 13 references

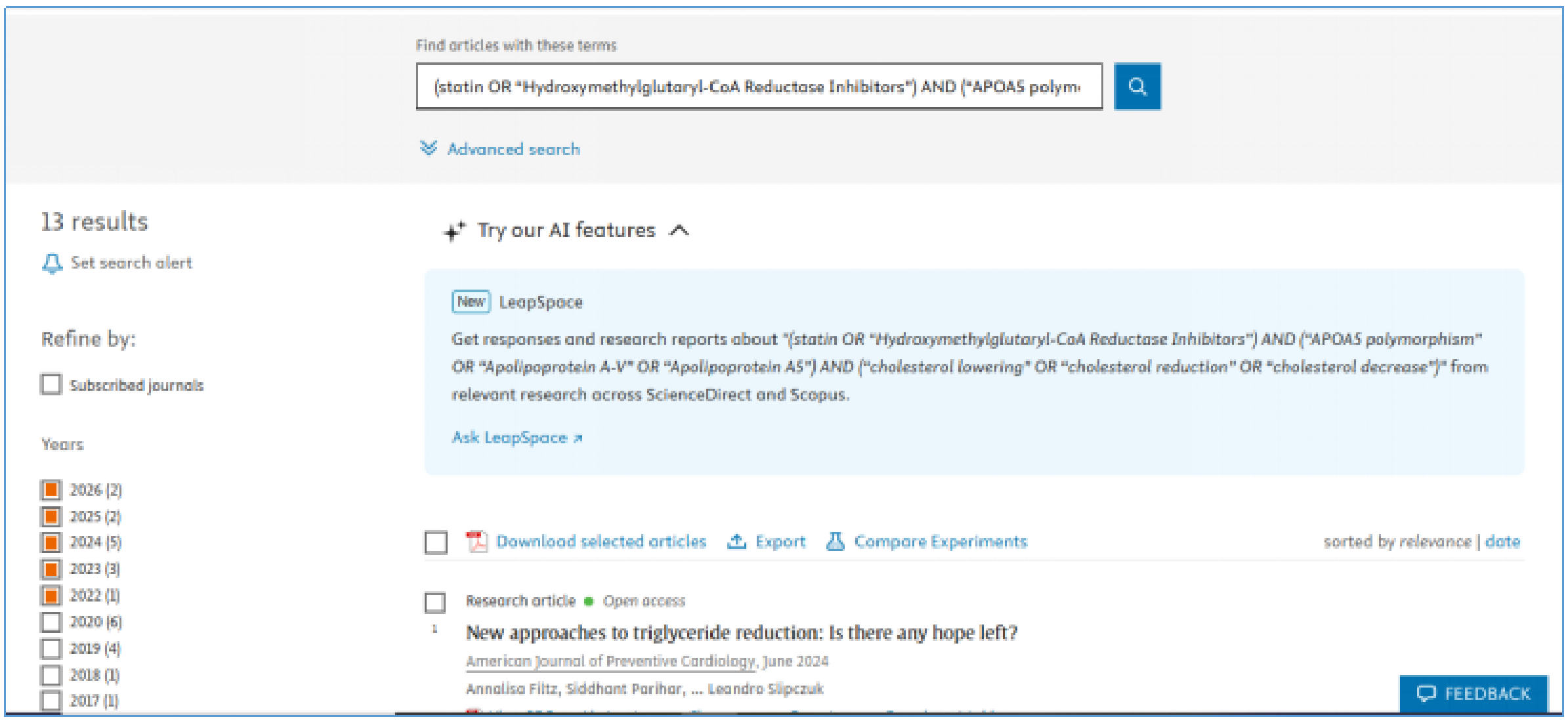

Supplement: Supplementary file 1 [file MJMS_33-3_Art2_Supplementary.pdf]
